# Metabolic regulation by prostaglandin E_2_ impairs lung group 2 innate lymphoid cell responses

**DOI:** 10.1111/all.15541

**Published:** 2022-10-14

**Authors:** Calum T. Robb, You Zhou, Jennifer M. Felton, Birong Zhang, Marie Goepp, Privjyot Jheeta, Danielle J. Smyth, Rodger Duffin, Sonja Vermeren, Richard M. Breyer, Shuh Narumiya, Henry J. McSorley, Rick M. Maizels, Jürgen K. J. Schwarze, Adriano G. Rossi, Chengcan Yao

**Affiliations:** ^1^ Centre for Inflammation Research, Queen's Medical Research Institute The University of Edinburgh Edinburgh UK; ^2^ Systems Immunity University Research Institute and Division of Infection and Immunity Cardiff University Cardiff UK; ^3^ Division of Cell Signaling and Immunology, School of Life Sciences Wellcome Trust Building, University of Dundee Dundee UK; ^4^ Department of Veterans Affairs Tennessee Valley Health Authority Nashville Tennessee USA; ^5^ Department of Medicine Vanderbilt University Medical Center Nashville Tennessee USA; ^6^ Alliance Laboratory for Advanced Medical Research and Department of Drug Discovery Medicine, Medical Innovation Center Kyoto University Graduate School of Medicine Kyoto Japan; ^7^ Wellcome Centre for Molecular Parasitology, Institute for Infection, Immunity and Inflammation University of Glasgow Glasgow UK

**Keywords:** cellular metabolism, group 2 innate lymphoid cell (ILC2), lung allergy, NSAID‐exacerbated respiratory disease, prostaglandin E_2_

## Abstract

**Background:**

Group 2 innate lymphoid cells (ILC2s) play a critical role in asthma pathogenesis. Non‐steroidal anti‐inflammatory drug (NSAID)‐exacerbated respiratory disease (NERD) is associated with reduced signaling via EP2, a receptor for prostaglandin E_2_ (PGE_2_). However, the respective roles for the PGE_2_ receptors EP2 and EP4 (both share same downstream signaling) in the regulation of lung ILC2 responses has yet been deciphered.

**Methods:**

The roles of PGE_2_ receptors EP2 and EP4 on ILC2‐mediated lung inflammation were investigated using genetically modified mouse lines and pharmacological approaches in IL‐33‐induced lung allergy model. The effects of PGE_2_ receptors and downstream signals on ILC2 metabolic activation and effector function were examined using in vitro cell cultures.

**Results:**

Deficiency of EP2 rather than EP4 augments IL‐33‐induced mouse lung ILC2 responses and eosinophilic inflammation in vivo. In contrast, exogenous agonism of EP4 and EP2 or inhibition of phosphodiesterase markedly restricts IL‐33‐induced lung ILC2 responses. Mechanistically, PGE_2_ directly suppresses IL‐33‐dependent ILC2 activation through the EP2/EP4‐cAMP pathway, which downregulates STAT5 and MYC pathway gene expression and ILC2 energy metabolism. Blocking glycolysis diminishes IL‐33‐dependent ILC2 responses in mice where endogenous PG synthesis or EP2 signaling is blocked but not in mice with intact PGE_2_‐EP2 signaling.

**Conclusion:**

We have defined a mechanism for optimal suppression of mouse lung ILC2 responses by endogenous PGE_2_‐EP2 signaling which underpins the clinical findings of defective EP2 signaling in patients with NERD. Our findings also indicate that exogenously targeting the PGE_2_‐EP4‐cAMP and energy metabolic pathways may provide novel opportunities for treating the ILC2‐initiated lung inflammation in asthma and NERD.

## Graphical abstract

Deficiency of EP2 rather than EP4 enhances IL‐33‐induced lung ILC2 responses and eosinophilic inflammation. Both EP2 and EP4 agonism represses IL‐33‐induced mouse lung ILC2 responses. PGE_2_‐EP2/EP4‐cAMP signaling inhibits ILC2 survival, proliferation and effector function through disturbing the IL‐2/IL‐7/IL‐33‐activated metabolic program in ILC2s.Abbreviations: AMP, adenosine monophosphate; cAMP, cyclic adenosine monophosphate; EP2, E prostanoid receptor 2; EP4, E prostanoid receptor 4; ILC2, group 2 innate lymphoid cell; NF‐κB, nuclear factor kappa B; PDE, phosphodiesterase; PGE_2_, prostaglandin E_2_; STAT5: signal transducer and activator of transcription 5.

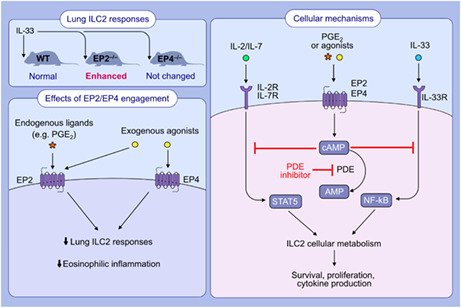

**Abbreviations**
cAMP: cyclic adenosine monophosphate
EP2: E prostanoid receptor 2
EP4: E prostanoid receptor 4
FSC: forward scatter
IL: interleukin
ILC2: group 2 innate lymphoid cells
NERD: NSAID‐exacerbated respiratory disease
NSAID: non‐steroidal anti‐inflammatory drugs
PDE: phosphodiesterase
PG: prostaglandin
PGE_2_
: prostaglandin E_2_
STAT5: signal transducer and activator of transcription 5
Th2: type 2 helper T cells

## 1 INTRODUCTION

Asthma is a chronic inflammatory lung disease characterized by bronchoconstriction and airway hyperresponsiveness. Upon contact with allergens, irritants (e.g., pollen, air pollutants) or infections, the damaged lung epithelial cells release pro‐allergic cytokines such as interleukin (IL)‐33, which rapidly activates many immune cells including group 2 innate lymphoid cells (ILC2s). ILC2s are innate lymphocytes without antigen‐specific receptors, but they highly express type 2 helper (Th2) transcription factors such as GATA3 and epithelial cytokine receptors including ST2, a receptor for IL‐33.^1^ In response to stimuli by epithelial cytokines, ILC2s produce large amounts of type 2 cytokines (e.g., IL‐5, IL‐13), which initiate the early onset of innate allergic inflammation.^1^ Lack or reduction of ILC2s leads to not only decline of type 2 inflammation during diseases, as found in asthma, metabolic diseases and cancer, but also alteration of type 2 immunity following parasite infections.^1^, ^2^ ILC2s also contribute to Th2 cell activation, sustaining chronic allergic inflammation.^3^, ^4^ ILC2s have been reported to present in human lungs and their cytokine production is associated with disease severity,^5^, ^6^, ^7^ but a possibility that those ILC2s found in human lungs may be contaminated from peripheral blood cannot be ruled out. Furthermore, ILC2s and their cytokine production (e.g., IL‐13) are increased in inflamed sinonasal mucosa from chronic rhinosinusitis with nasal polyps,^8^ a condition that is associated with hypersensitivity to aspirin.

Eicosanoids are bioactive lipid mediators that play critical roles in regulation of type 2 immune responses and allergic diseases.^9^ Non‐steroidal anti‐inflammatory drugs (NSAIDs) such as aspirin, ibuprofen, and indomethacin are widely used for anti‐pyretic and analgesic purposes during acute and chronic inflammation through blocking biosynthesis of prostaglandins (PGs) including PGE_2_.^10^ However, hypersensitivity reactions to NSAIDs can occur, causing NSAID‐exacerbated respiratory disease (NERD), a chronic type 2 immune mediated respiratory disease linked to asthma and nasal polyposis.^11^ Increased ILC2 numbers have been observed in the nasal mucosa of patients with NERD,^12^ indicating a role for ILC2s in NERD pathogenesis.^13^ NERD patients exhibit imbalanced arachidonic acid metabolism with overproduction of PGD_2_ and leukotrienes (e.g., LTD_4_ and LTB_4_) but reduction of PGE_2_.^14^ PGD_2_ and leukotrienes promote ILC2 recruitment to the lung and cytokine production.^15^, ^16^ Prostacyclin (also called PGI_2_) has been reported to restrict ILC2 activation and effector function.^17^ NERD patients have reduced expression of the *PTGES* gene (encoding the key PGE_2_ synthase, mPGES1) due to hypermethylation.^18^ Genetic studies have also suggested that polymorphisms in the *PTGER2* gene (which encodes PGE_2_ receptor EP2) are specifically associated with aspirin‐intolerant asthma.^19^ Moreover, immune cells from the NERD patients display reduced EP2 expression.^14^, ^20^ These clinical studies suggest that lessening of PGE_2_‐EP2 signaling may be a causative factor in the development of NERD, but the underlying mechanisms remain to be elucidated.

PGE_2_ plays distinct roles during the sensitization and challenge stages of T cell‐mediated allergic inflammation, possibly via both EP2 and EP4.^21^ PGE_2_ through EP4 suppresses neutrophilic lung inflammation in ex vivo human cell culture systems and various animal models.^22^, ^23^, ^24^ Lung allergic responses were increased in mice deficient in PGE_2_ synthases such as COX2.^25^ Recently, it was reported that PGE_2_ suppressed human ILC2 function in vitro through its receptors EP2 and EP4.^26^ However, the respective effects of EP2 and EP4 on ILC2‐mediated type 2 immune responses in vivo remain unclear. Although EP4 deficiency in hematopoietic cells was reported to enhance ILC2 responses in mouse,^27^ some critical questions remain to be elucidated. For example, what is the role of endogenous EP2 signaling in the control of lung ILC2 responses, if exogenous activation of PGE_2_ receptor signaling helps control of lung ILC2 responses, and what this means to human NERD. Thus, it is imperative to properly assess the actions of PGE_2_ receptors on lung ILC2 responses in vivo. Here, we investigate the impact of endogenous versus exogenous activation of EP2 and EP4 on modulation of ILC2‐mediated type 2 immune responses in the mouse lung using gene‐modified mouse models and pharmacological approaches.

## 2 METHODS

### 2.1 Mice

*Rag2* ^−/−,^ ^28^ EP2(*Ptger2*)^−/−,^ ^29^ EP4(*Ptger4*)^−/−^ ^30^ mice were maintained under specific pathogen‐free conditions in accredited animal facilities in the University of Edinburgh. Vav^Cre^EP4^fl/fl^ and Thy1.2^iCre^EP4^fl/fl^ mice were generated by crossing Vav‐Cre mice^31^ and Thy1.2^iCre^ mice,^32^ respectively, to EP4‐floxed mice^33^ and maintained under specific pathogen‐free conditions in accredited animal facilities in the University of Edinburgh. All the genetically modified mouse strains are at the C57BL/6 background. Wild‐type female C57BL/6 mice were purchased from Harlan UK or bred in animal facilities within the University of Edinburgh. Sex‐matched mice aged >6 weeks were used in experiments. Mice were randomly allocated into different treatment groups and analysed individually. No animal was excluded for analysis except one mouse in Figure 2A which was overlooked at the step of ex vivo restimulation of lung single cells with PMA, ionomycin and Brefeld A (for performing intracellular cytokine staining). All animal experiments were conducted in accordance with the U.K. Animals (Scientific Procedures) Act of 1986 with local institutional ethical approval by the University of Edinburgh Animal Welfare and Ethical Review Body.

### 2.2 Reagents

Antibodies to mouse CD45 (clone 30‐F11), CD19 (clone 6D5), NK‐1.1 (clone PK136), CD4 (clone RM4‐5), CD90.2 (clone 30‐H12), CD3e (clone 145‐2C11), CD11b (clone M1/70), CD11c (clone N418), ICOS (clone7E.17G9), IL‐5 (clone TRFK5), IL‐13 (clone eBio13A), Ki‐67 (clone 16A8) and Ly6G (clone 1A8) were purchased from eBioscience or Biolegend. Antibodies to mouse ST2(IL33‐R) (clone DJ8) and SiglecF (clone E50‐2440) were purchased from mdbioproducts and BD respectively. LIVE/DEAD™️ Fixable Blue Dead Cell Stain Kit for UV excitation, UltraComp eBeads™️ and mouse IL‐5/IL‐13 ELISA kits were purchased from Thermo Fisher Scientific. Recombinant mouse IL‐33 were purchased from Biolegend. PGE_2_, Butaprost (free acid) and L‐902688 (EP4 agonist) were obtained from Cayman, while PF‐04418948 was purchased from Abcam. Indomethacin, phorbol myristate acetate (PMA), ionomycin, brefeldin A, dibutyryl cyclic adenosine monophosphate (db‐cAMP) and 3‐isobutyl‐1‐methyxanthine (IBMX) were purchased from Sigma. Complete RMPI consisted of RPMI 1640 (Gibco) supplemented with 10% FBS, 1% Penicillin/Streptomycin, 1% L. glutamine and 50 μM β‐mercaptoethanol.

### 2.3 Airway administration of pro‐allergic cytokines

**FIGURE 1:**
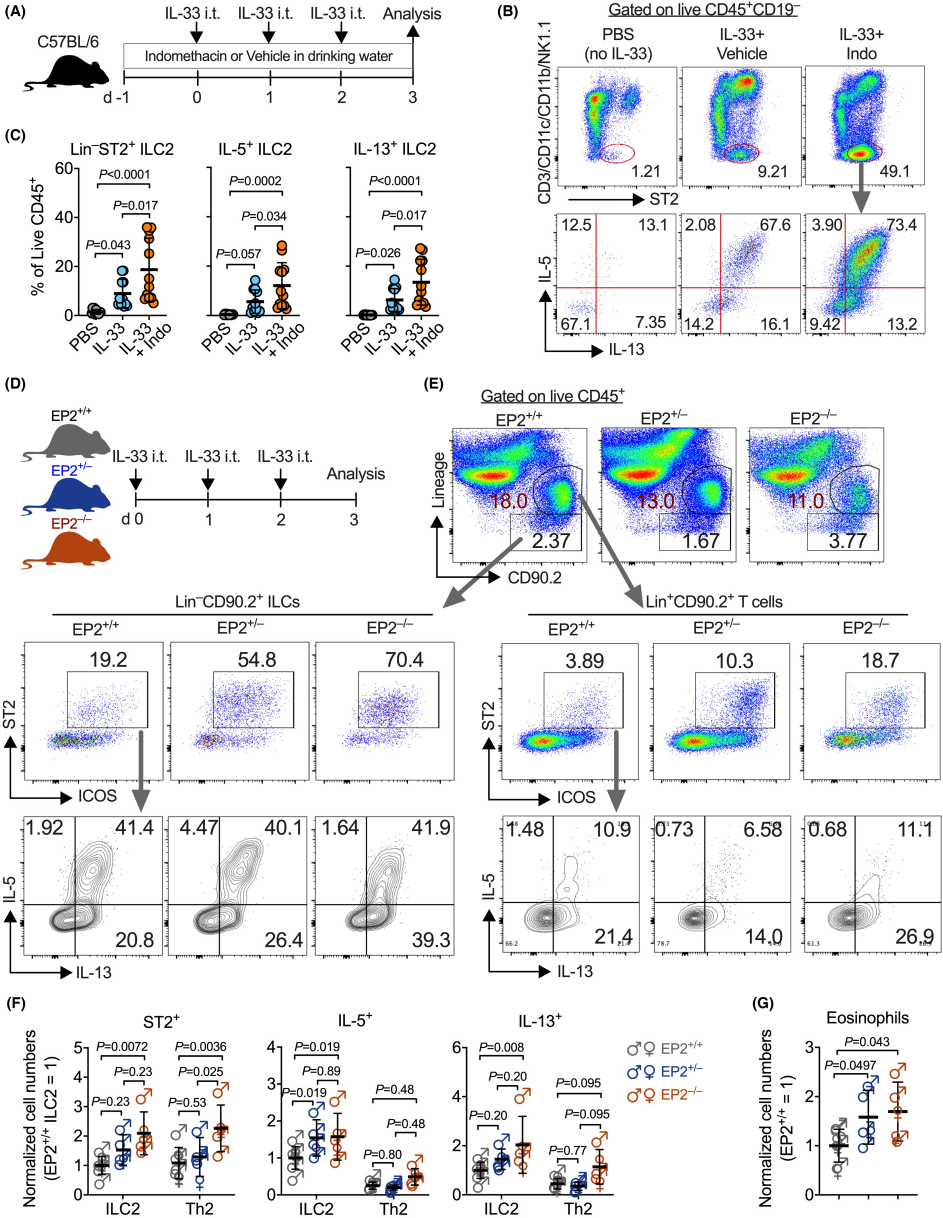
EP2 deficiency augments IL‐33‐induced lung ILC2 responses. (A) Experimental design. Female C57BL/6 mice were intratracheally administered IL‐33 for 3 consecutive days and received indomethacin or control in drinking water, and mice were sacrificed 24 h after the last IL‐33 challenge. PBS (*n* = 11), IL‐33 with vehicle (*n* = 11) or indomethacin (*n* = 12). (B and C) Representative flow cytometric dot plots (B) and collective percentages (C) of lung ILC2s. Cells in (B) were pre‐gated on live CD45^+^CD19^−^ immune cells. (D) EP2^+/+^ (*n* = 8) and EP2^+/−^ (*n* = 5) or EP2^−/−^ (*n* = 5) mice were intratracheally administered IL‐33 for 3 consecutive days and sacrificed 24 h after the last IL‐33 challenge. (E) Representative flow cytometric dot plots of lineage(CD3/CD19/CD11b/CD11c/NK1.1)^−^CD19^−^CD90.2^+^ST2^−^ ILC2s and lineage^+^CD90.2^+^ST2^+^ Th2 cells and IL‐5 versus IL‐13 expression in the lungs. Cells were pre‐gated on live CD45^+^ immune cells. (F and G) Number of ST2^+^ total ILC2s, IL‐5^+^ ILC2s, IL‐13^+^ ILC2s, and eosinophils in the lung. Data were normalized by sexes and presented as fold change to the EP2^+/+^ group in respective experiments. Data shown as means ± SDs were pooled from three independent experiments. Each dot in the bar graphs represents one mouse. *p* Values were calculated by one‐way ANOVA with post‐hoc Holm–Sidak's multiple comparisons tests. ns, not significant.

**FIGURE 2:**
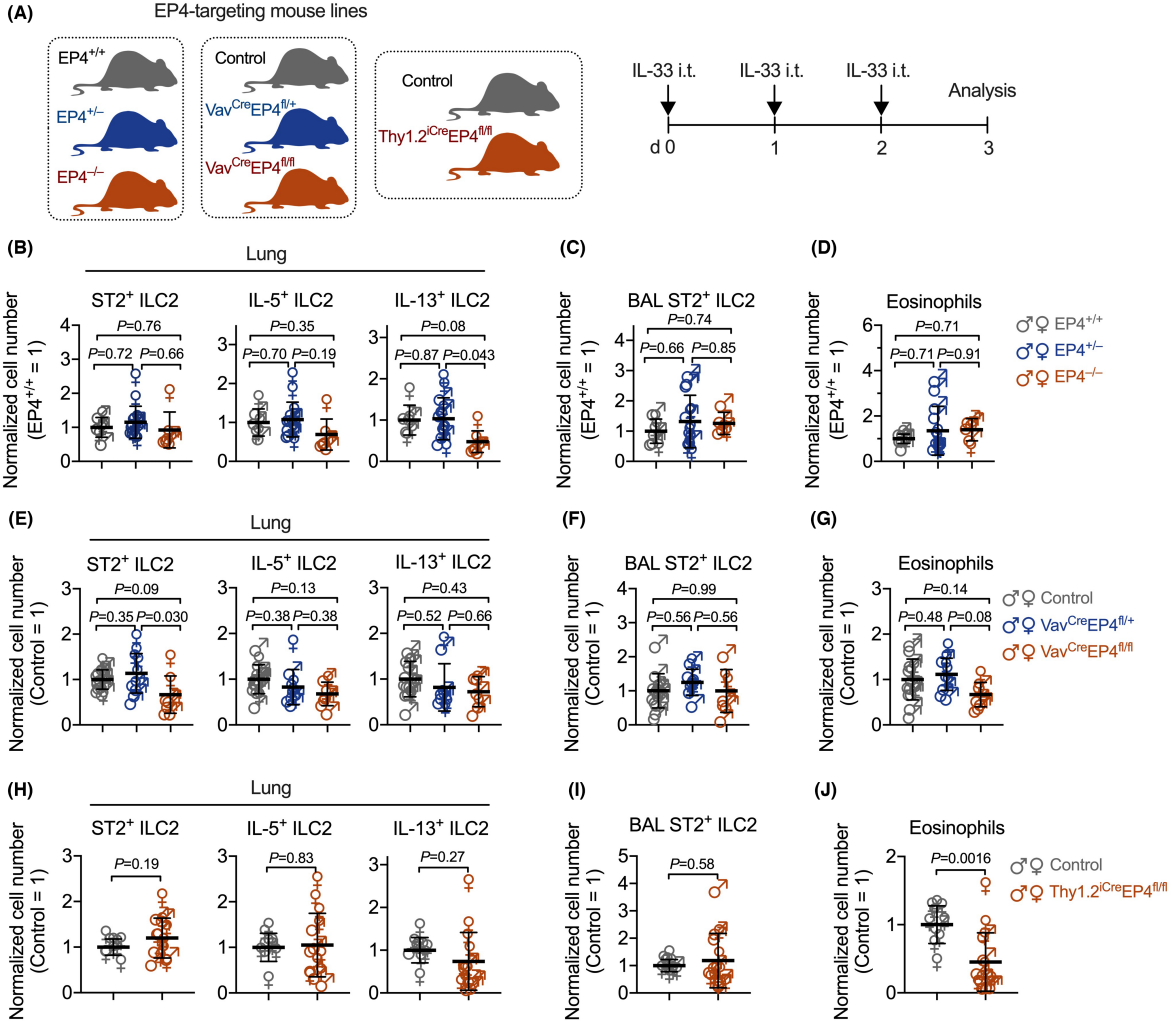
Endogenous EP4 signaling is dispensable for the control of IL‐33‐induced lung ILC2 responses. (A) Experimental design. Mice with various EP4 genotypes were intratracheally administered IL‐33 for 3 consecutive days and sacrificed 24 h after the last IL‐33 challenge. (B–D) Number of lung ST2^+^ total ILC2s, IL‐5^+^ ILC2s and IL‐13^+^ ILC2s (B), BAL ST2^+^ ILC2s (C) and eosinophils (D) in EP4^+/+^ (*n* = 7), EP4^+/−^ (*n* = 14) or EP4^−/−^ (*n* = 6) mice. (E–G) Number of lung ST2^+^ total ILC2s, IL‐5^+^ ILC2s and IL‐13^+^ ILC2s (E), BAL ST2^+^ ILC2s (F) and eosinophils (G) in control (EP4^fl/fl^ or EP4^fl/+^, *n* = 14), Vav^Cre^EP4^fl/+^ (*n* = 10), or Vav^Cre^EP4^fl/fl^ (*n* = 7) mice. (H–J) Number of lung ST2^+^ total ILC2s, IL‐5^+^ ILC2s and IL‐13^+^ ILC2s (H), BAL ST2^+^ ILC2s (I), and eosinophils (J) in control (EP4^fl/fl^ or EP4^fl/+^, *n* = 10) or Thy1.2^iCre^EP4^fl/fl^ (*n* = 14) mice. Data were normalised by sexes and presented as fold changes to the EP4^+/−^ (B–D) or control (E–J) groups, respectively. Data shown as means ± SDs were pooled from three independent experiments. Each dot in the bar graphs represents one mouse. *p* Values were calculated by one‐way ANOVA with post‐hoc Holm–Sidak's multiple comparisons tests (B–G) or unpaired, 2‐tailed Student's *t*‐test (H–J). ns, not significant.

**FIGURE 3:**
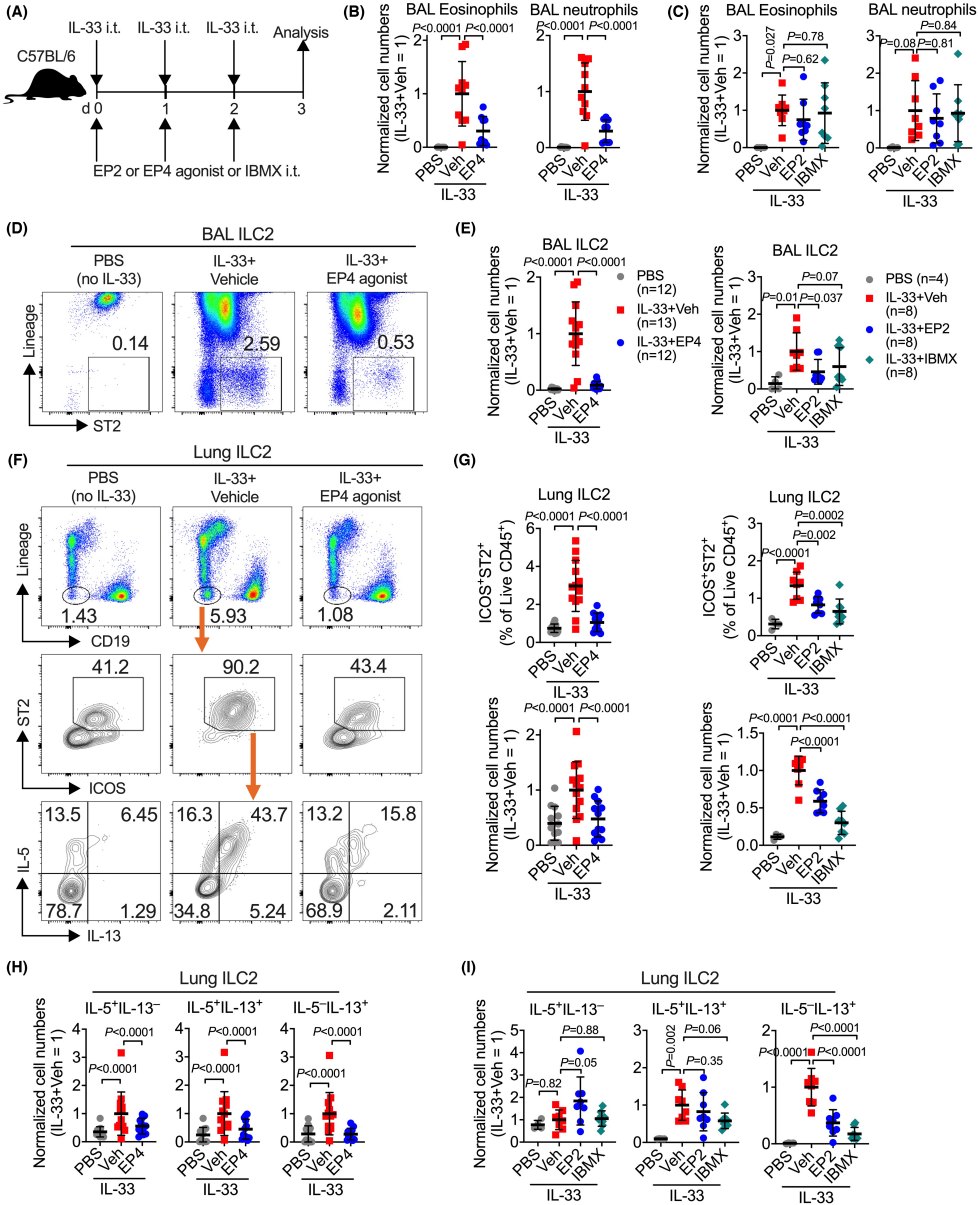
EP2 and EP4 agonism inhibits the alarmin IL‐33‐activated lung ILC2 responses. (A) Experimental design. Female C57BL/6 mice were administered intratracheally with PBS or IL‐33 with vehicle, EP2 agonist [butaprost (free acid)], EP4 agonist (L‐902688), or IBMX for 3 consecutive days, and sacrificed 24 h after the last IL‐33 challenge. (B and C) Eosinophils and neutrophils in the lungs and the bronchoalveolar lavages (BAL) fluids. (D and E) Representative flow cytometric dot plots and collective numbers of lineage(CD3/CD19/CD11c/CD11b/NK1.1)^−^ST2^+^ ILC2s in the BAL fluid. Cells in (D) were pre‐gated on the live CD45^+^ immune cells. (F) Representative flow cytometric dot plots of lineage(CD3/CD11c/CD11b/NK1.1)^−^CD19^−^, ST2^+^ ILC2s, and IL‐5‐ versus IL‐13‐expressing ILC2s in the lungs. Cells were pre‐gated on the live CD45^+^ immune cells. (G–I) Collective percentages and numbers of ST2^+^ total ILC2s and IL‐5‐ or IL‐13‐expressing ILC2s in the lungs. Data were normalised to the IL‐33 plus Vehicle groups in respective experiments, and shown as means ± SDs were pooled from 2–3 independent experiments. Each dot in the bar graphs represents one mouse. *p*‐values were calculated by one‐way ANOVA with post‐hoc Holm–Sidak's multiple comparisons tests. ns, not significant.

A variety of different mouse lines including C57BL/6 wild‐type, EP4^−/−^, Vav^Cre^EP4^fl/fl^, and their appropriate control mice were anaesthetised by inhalation of isoflurane and the pro‐allergic cytokine IL‐33 (200 ng per treatment) was administered via intratracheal administration once daily for consecutive 3 days. When indicated, EP2 or EP4 agonist (each 10 μg) or vehicle control (PBS) were co‐administered with IL‐33. Indomethacin (5 mg/kg/day), PF‐04418948 (10 mg/kg/day) or vehicle (0.5% EtOH) was administered in drinking water started one day before IL‐33 administration. 2‐Deoxy‐d‐glucose (2‐DG, 1 g/kg/day) (Abcam) was administered via intraperitoneal injection (one injection per day throughout the whole experiment). All animals were culled via anaesthetic overdose by i.p injection of 200 μl Pentoject Pentobarbital Sodium solution (200 mg/ml) and subsequent exsanguination. Bronchoalveolar lavage (BAL) was collected (x3 lavages with 800 μl ice cold PBS) followed by aseptic lung dissection for subsequent tissue digestion. Both male and female mice were used in Figures 1E–G, 2 and 5N,O, while only female WT mice were used in Figures 1B,C, 3, 5.

### 2.4 Murine lung and BAL single cell suspensions

The murine lungs were digested in Liberase TL (Roche) and DNase I (Sigma) at 37°C for 35 min. Digested tissue was passed through a 100 μm cell strainer and red blood cells lysed using ACK lysing buffer. Viable and dead lung cell counts were determined using 0.4% Trypan Blue solution and a TC10™ Automated Cell Counter (BioRad). BAL samples were centrifuged and supernatants from the first BAL retrieval harvested, snap frozen on dry ice and stored at −80°C for subsequent cytokine detection using mouse IL‐5/IL‐13 ELISA kits according to manufacturer's instructions. Red blood cells were lysed from BAL cell pellets using ACK lysing buffer and cell counts performed as before.

### 2.5 ILC2 in vitro culture

For in vitro experiments, all the work was carried out aseptically and single cell suspensions were obtained from lungs and bone marrow of untreated *Rag2* ^−/−^ mice. Whole lung and bone marrow cells were cultured in complete RPMI with a cytokine cocktail (IL‐2, IL‐7, and IL‐33) with various reagents indicated in the figure legends at 37°C, 5% CO_2_ for 3–4 days. IL‐5 and IL‐13 in the supernatants were analysed by ELISA. In some experiments, CD90.2^+^ cells were pre‐sorted from *Rag2* ^−/−^ lung and bone marrow single cell suspensions using an EasySep™ Mouse CD90.2 Positive Selection Kit II (Stemcell Technologies) according to manufacturer's instructions. Live Lineage(CD11c/CD11b/NK1.1)^−^CD45^+^CD90.2^+^ST2^+^ ILC2s were sorted using a BD FACS Aria II and then cultured in complete RPMI containing 50 μM β‐mercaptoethanol with IL‐2, IL‐7, and IL‐33 with or without PGE_2_ in a round bottom 96‐well plate at 37°C in 5% CO_2_ for 3 days.

### 2.6 Flow cytometry

For surface staining only, lung and BAL single cell suspensions were washed in PBS and stained with LIVE/DEAD™️ Fixable Blue Dead Cell Stain Kit. Cells were then blocked with IgG from rat serum and surfaced stained. For lung and BAL neutrophil/eosinophil detection, cell suspensions were labelled with CD45, CD11b, CD11c, Ly6G, and SiglecF. For BAL ILC2 detection, cell suspensions were labelled with a cocktail of lineage negative (Lin‐) markers consisting of CD3e, CD19, CD11b, CD11c and NK1.1, as well as CD45, CD4, CD90.2, ICOS, and ST2. In some experiments, anti‐mouse CD200R3 Ab was added to the lineage marker list. For intracellular staining, single cell lung suspensions were stimulated with a cocktail of PMA, Ionomycin and Brefeldin A (or Golgiplug) and cultured in complete RPMI for 4 h at 37°C with 5% CO_2_. After stimulation, lung cells were Live/Dead stained, blocked and surface stained for ILC2s as before. Next, lung cells were fixed with intracellular fixation buffer (eBioscience), and then stained for intracellular IL‐5, IL‐13 and Ki‐67 for 20 min at 4°C. To measure c‐Myc expression and STAT5 and S6 phosphorylation, lung and bone marrow CD90.2+ cells from naïve Rag2−/− mice were cultured with IL‐2, IL‐7 and IL‐33 forr 7–10 days, and then live CD45 + Lin‐ST2+ ILC2s were sorted using flow cytometry and cultured with or without PGE_2_ or db‐cAMP in the presence or absence of the cytokine cocktail (IL‐2/IL‐7/IL‐33) for further 3 days. Cells were fixed and stained with antibodies against c‐Myc (clone E5Q6W), pSTAT5 (clone C71E5), or pS6 (clone C71E5, all from Cell Signaling Technology) using the Foxp3 staining kit (eBioscience). Cell samples were acquired on a BD LSR Fortessa analyser, and results were analysed by FlowJo™.

### 2.7 Seahorse assays

The Seahorse XFe96‐well metabolic analyser (Agilent) was used to investigate the effect PGE_2_ may have upon ILC2 glycolysis in vitro. *RAG2* ^*−/−*^ mice were subjected to i.t administrations of IL‐33 (one i.t. per mouse per day) on days 0, 2 and 4. Lung and BAL single cell suspensions were prepared on day 6 and pooled together for sorting ILC2s as described above. 4 × 10^4^ ILC2s were rested overnight in a 96‐well round bottom plate in complete RPMI (supplemented with L‐glutamine) and IL‐2 and IL‐7 (both 10 ng/ml). On the next day, IL‐33 (10 ng/ml), PGE_2_ (1 μM) or DMSO (0.01% v/v as vehicle) was added to media and cultured for a further 24 h. The next day, ILC2 counts were performed on each treatment to confirm >95% viability. Approximately 4 × 10^4^ ILC2s re‐suspended in 50 μl Seahorse XF RPMI pH 7.4 (Agilent) (+1 M glucose, 100 mM pyruvate and 200 mM glutamine) were added in triplicate to wells of a Seahorse XF96 V3 PS cell culture microplate (Agilent) coated 24 h prior with Cell‐Tak™️ cell adhesive (Sigma). Precise measurements of glycolysis in ILC2s were carried out using the Seahorse XF Glycolytic Rate Assay Kit (Agilent; 103344‐100) according to manufacturer's instructions. The real time oxygen consumption rate (OCR), extracellular acidification rate (ECAR), and proton efflux rate (PER) of ILC2s during glycolysis was directly measured, including glycolytic rates for basal conditions and compensatory glycolysis after injections of 0.5 μM Rotenone/Antimycin A (inhibits mitochondrial respiration) and 50 mM 2‐DG (inhibits glycolysis) respectively into all treatment wells.

### 2.8 RNA‐sequencing data analysis

To explore the effect of cAMP on ILC2 gene expression, we first downloaded raw sequence data in FASTQ format from the GEO database (accession number: GSE131996). FastQC was used to assess the quality of FASTQ files (https://www.bioinformatics.babraham.ac.uk/projects/fastqc/). Sequence reads were mapped to mouse genome using STAR 2.7.^34^ QualiMap^35^ was used to assess the quality of mapped data and featureCounts was employed to count uniquely mapped fragments against genomic features defined by the GENCODE annotation file (Mus_musculus.GRCm39.103.gtf). The counts were further processed for differential expression gene analysis in R (4.0.3). Differentially expressed genes were analysed by DESeq2^36^ and significance was identified using adjusted *p*‐value < .05 and the absolute value log2 fold change ≥1. Ensembl version 103 was used for gene annotation. Gene Set Enrichment Analysis^37^ was performed for functional analysis using the Molecular Signatures Database v7.4. Hallmark, KEGG and REACTOME curated gene sets were used as references for screening enriched pathways. *p* values were adjusted and enrichment scores were normalised. An adjusted *p*‐value < .05 (Benjamini–Hochberg) was used as an indicator of significance in pathway analysis.

### 2.9 Statistical analyses

All data were expressed as mean ± SD (in vivo) or SEM (in vitro). For certain experiments where both sexes of animals were used, absolute cell numbers were normalised by sex and data were presented as fold changes. Statistical significance between two groups was examined using an unpaired, 2‐tailed Student's *t*‐test. One‐way and two‐way ANOVA with post‐hoc Holm–Sidak's multiple comparisons tests were used to evaluate statistical significance between multiple groups. Statistical analyses were performed using Prism 8 software (Graphpad) and significance was accepted at *p* < .05.

## 3 RESULTS

### 3.1 Inhibition of endogenous PG synthesis augments IL‐33‐dependent lung ILC2 responses

At the steady state, PGE_2_ is expressed in most tissues including the lung and its levels are elevated by inflammatory stimuli. We thus investigated whether endogenous PGE_2_ suppressed lung ILC2 immune responses. We administered intratracheally IL‐33 into the lungs of wild‐type (WT) C57BL/6 mice. Mice also received indomethacin, an NSAID that blocks the biosynthesis of all PGs including PGE_2_, or vehicle control in drinking water (Figure 1A). In agreement with a previous report,^38^ indomethacin significantly increased IL‐33‐mediated accumulation of CD45^+^Lineage(Lin)^−^ST2^+^ ILC2s to the lung and enhanced type 2 cytokine (i.e., IL‐5 and IL‐13) production from ILC2s (Figure 1B,C). However, indomethacin treatment had no effect on the accumulation of eosinophils in the lung (data not shown), possibly because indomethacin inhibits all PG synthesis and some PGs have direct actions on eosinophils. These results suggest that endogenous PGs suppress lung ILC2 responses.

### 3.2 EP2 deficiency enhances IL‐33‐induced lung ILC2 responses

As indomethacin inhibits biosynthesis of all PGs, we examined whether PGE_2_ is involved in the regulation of lung ILC2 responses, and if yes, through which receptor(s). We examined published datasets^39^ and found that ILC2s isolated from various tissues including lung, bone marrow, skin, and intestine have considerably higher gene expression of EP4, followed by EP2 and EP1, and that EP3 has much lower expression levels in ILC2s (Figure S1). Given that down‐regulation of EP2 is associated with NERD,^14^ we first asked whether blockade of endogenous PGE_2_‐EP2 signaling influenced lung ILC2 responses. We injected IL‐33 into the lungs of EP2‐deficient^29^ or control mice and measured lung ILC2 activation (Figure 1D). As expected, EP2‐deficiency increased lung ILC2 accumulation and production of type 2 cytokines (IL‐5 and IL‐13) in response to IL‐33 (Figure 1E,F), although naïve EP2 deficient mice had similar lung ILC2s compared to naive WT mice (Figure S2A,B). EP2 (*Ptger2*) gene expression was reduced by half in EP2‐heterozygous mice (Figure S2C), which led to increased IL‐5‐producing ILC2s but did not affect ST2^+^ total ILC2s or IL‐13‐producing ILC2s (Figure 1F), indicating that EP2 expression levels may differently control distinct ILC2 subpopulations. EP2 deficiency also increased lung Lin^+^CD90.2^+^ST2^+^ Th2 cells, but type 2 cytokine production from T cells was not significantly affected by the loss of EP2 (Figure 1E,F). In agreement with the increase in ILC2s, EP2‐deficient mice had more eosinophils after IL‐33 treatment (Figure 1G and Figure S3). Our data suggest that endogenous PGE_2_‐EP2 signaling restricts IL‐33‐dependent lung ILC2 responses.

### 3.3 EP4 deficiency does not affect IL‐33‐induced lung ILC2 responses

To test whether blocking PGE_2_‐EP4 signaling modulates lung ILC2 responses, we administered IL‐33 to global EP4‐deficient^30^ and control mice (Figure 2A). Unexpectedly, EP4 deficiency did not enhance ILC2 accumulation in the lung but reduced type 2 cytokine production compared to WT and heterozygous mice (Figure 2B,C). Global EP4 deficiency also did not increase eosinophils in the lung (Figure 2D). We then investigated whether specific deletion of EP4 in immune cells including ILC2s enhances lung ILC2 responses. As there were no mouse lines that selectively target only ILC2s, we generated Vav^Cre^EP4^fl/fl^ mice by crossing EP4‐floxed mice^33^ to Vav‐cre mice^31^ which drives ablation of EP4 in all hematopoietic‐derived immune cells (including ILC2s) (Figure S4A). Naïve Vav^Cre^EP4^fl/fl^ mice had similar lung ILC2s to naïve control EP4^fl/fl^ mice (Figure S4B,C). A previously published report showed that Vav‐dependent EP4 deficiency enhanced lung ILC2 function and eosinophilic inflammation.^27^ However, we found that mice with conditional EP4 deficiency in all immune cells had comparable lung ILC2 responses after IL‐33 administration, albeit with a moderate reduction of lung ST2^+^ ILC2 accumulation, compared to control EP4^fl/fl^ and Vav^Cre^EP4^fl/+^ mice (Figure 2E,F). Homozygous Vav^Cre^EP4^fl/fl^ mice had a trend to reduce eosinophils (Figure 2G), which was likely due to a direct effect of EP4 on Vav‐expressing eosinophils. Both EP4^−/−^ and Vav^Cre^EP4^fl/fl^ mice ablated EP4 from the germline, which may have a potential effect on ILC (including ILC2) development. To exclude this possibility, we employed an inducible EP4 deficient mouse line by crossing Thy1.2^iCre^ mice^32^ to EP4‐floxed mice to generate Thy1.2^iCre^EP4^fl/fl^ mice, which do not have EP4 expression in Thy1.2‐expressing cells (i.e., all ILC and T cells) after administration of tamoxifen. Again, Thy1.2‐iCre‐driven inducible EP4 deficiency in lymphocytes did not alter IL‐33‐induced lung ILC2 responses (Figure 2H,I). Thy1.2‐iCre‐driven inducible EP4 deficiency significantly reduced lung eosinophils (Figure 2H–J), which may be due to the effects of EP4 signaling on other Thy1.2‐expressing cells such as the neuron, stromal or endothelial cells. Thus, our results suggest that endogenous PGE_2_‐EP4 signaling does not suppress lung ILC2 responses.

### 3.4 EP2 and EP4 agonism inhibit IL‐33‐induced lung ILC2 responses

To examine the effects of exogenous activation of EP2 and EP4 on ILC2 responses in vivo, we intratracheally administered IL‐33 together with highly selective agonist for EP2 [butaprost (free acid)] or EP4 (L‐902688) into C57BL/6 mice (Figure 3A). Administration of IL‐33‐induced accumulation of eosinophils and neutrophils in the bronchoalveolar lavage (BAL), while such accumulation was almost completely attenuated by co‐administration of the EP4 agonist (Figure 3B). The EP4 agonist had no effects on IL‐33‐induced lung ILC2 responses in EP4KO mice (Figure S5), indicating that its inhibitory actions were not due to the off‐target effects. Co‐administration of an EP2 agonist had no significant effects on IL‐33‐dependent accumulation of eosinophils and neutrophils (Figure 3C). Flow cytometric analysis suggested that IL‐33‐induced recruitment of lineage‐negative ST2^+^ ILC2s in the airway was impeded by both EP4 and EP2 agonists (Figure 3D,E). Analysis of lung single cells showed that IL‐33 increased numbers of total ST2^+^ ILC2s and their production of type 2 cytokines (e.g., IL‐5 and IL‐13), with the accumulation of ILC2s was again inhibited by both EP4 and EP2 agonists (Figure 3F–I).

Engagement of EP2 and EP4 activates the intracellular cAMP pathway. To test if the elevation of cAMP contributes PGE_2_ suppression of lung ILC2 responses, we administered 3‐isobutyl‐1‐methylxanthine (IBMX), a non‐selective inhibitor of cyclic nucleotide phosphodiesterases (PDEs) that increases the intracellular cAMP level through blocking cAMP degradation. Like EP2 or EP4 agonists, IBMX significantly reduced lung ILC2 numbers and type 2 cytokine production (Figure 3E,G,I). It is worth to note that although the EP4 agonist markedly suppressed IL‐5 production from both IL‐5^+^IL‐13^−^ and IL‐5^+^IL‐13^+^ ILC2s in response to IL‐33 administration, neither the EP2 agonist nor IBMX reduced IL‐5 production from lung ILC2s (Figure 3H,I). This may be the cause for unchanged eosinophils by EP2 agonist and IBMX as eosinophil activation is driven by IL‐5. In addition, the recent studies suggested that platelet activation and platelet‐adherent leukocytes were associated with NERD,^40^, ^41^ which might link to disruption of eicosanoid balance such as overproduction of cysteinyl leukotriene and reduction of PGE_2_.^14^, ^41^ As platelet adherent promotes lung ILC2 effector function,^42^, ^43^ we examined whether PGE_2_‐EP2‐cAMP signaling had an effect on platelet‐adherent (i.e., CD41‐positive) ILC2s using flow cytometry. IL‐33 treatment indeed increased the numbers of CD41^+^ ILC2s in the lung, and this was abrogated by EP2 agonist and IBMX, although neither IL‐33 nor EP2 agonist or IBMX affected CD41 adherent at the single cell level (Figure S6). Thus, exogenously activated the PGE_2_‐EP2/EP4‐cAMP pathway suppresses lung ILC2 responses.

### 3.5 PGE_2_‐EP2/EP4‐cAMP signaling directly inhibits ILC2 activation

We next examined the underlying mechanisms for PGE_2_ suppression of ILC2 responses. To this end, we firstly stimulated whole lung cells isolated from Rag2^−/−^ mice (which have no T or B cells but have ILCs including ILC2s) in vitro with a cytokine cocktail including IL‐33, IL‐2 and IL‐7 for 3–5 days. This cytokine cocktail markedly increased viable CD45^+^ immune cells, expanded the Lin^−^ST2^+^ ILC2 population, and induced IL‐5 and IL‐13 production from ILC2s in the whole lung cell cultures (Figure S7A). As seen in human ILC2 cell cultures,^26^ addition of PGE_2_, EP2 and EP4 agonists significantly reduced the numbers of live Lin^−^ST2^+^ ILC2s and overall IL‐5 and IL‐13 production (Figure S7B,C). Among live ILC2s, PGE_2_ and EP2 agonist significantly inhibited IL‐5 and IL‐13 double positive cells but increased IL‐5 single expressing cells, while EP4 agonist did not influence cytokine production from live ILC2s (Figure S7B). These results suggest that PGE_2_ inhibits lung ILC2 cell activation and effector function.

**FIGURE 4:**
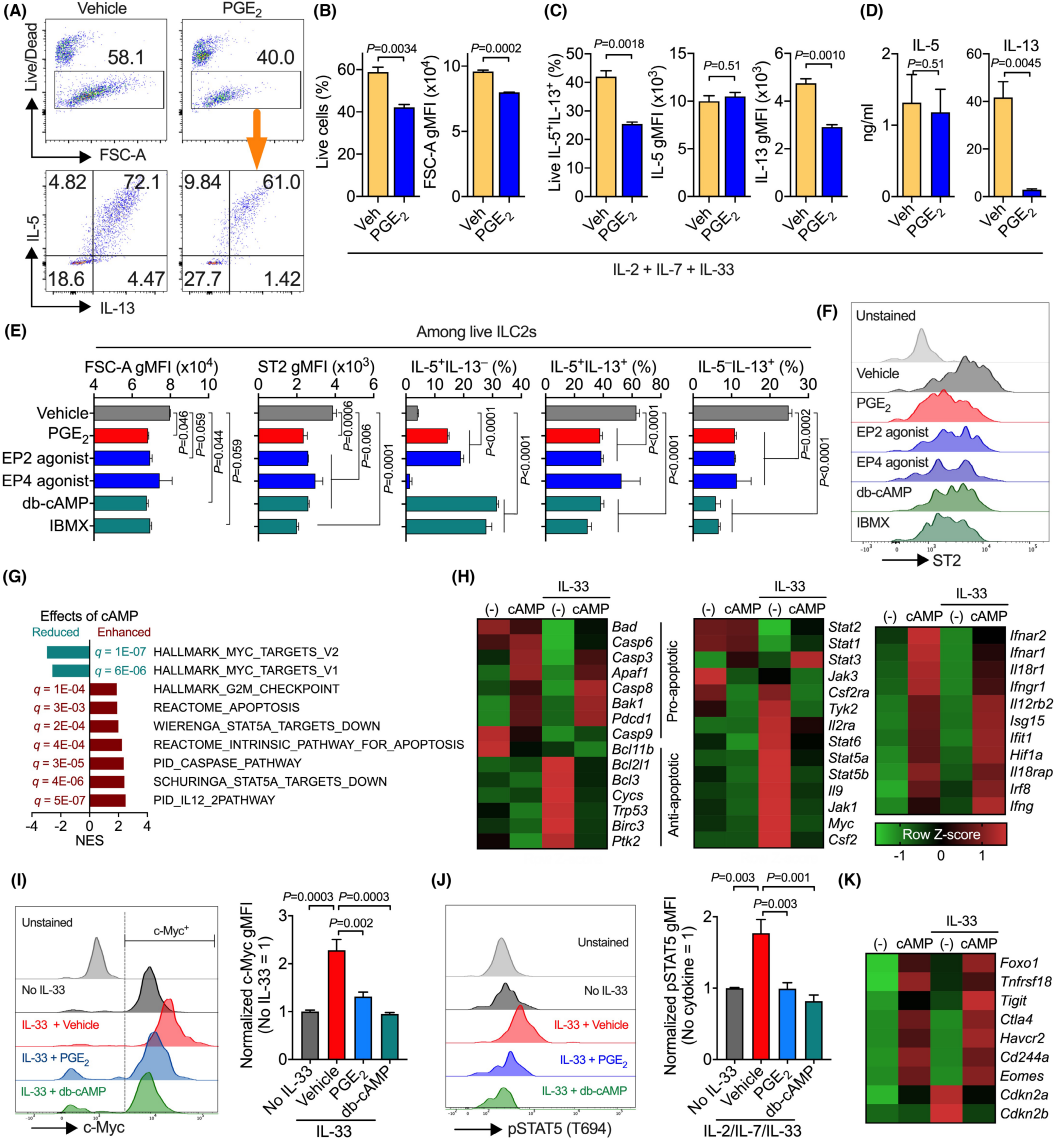
PGE_2_‐cAMP signaling directly inhibits ILC2 activation and alters ILC2 gene expression associated with cellular metabolic pathways. (A–D) Representative flow cytometric dot plots (A), cell viability and size (FSC‐A) (B), collective percentages of live IL‐5^+^IL‐13^+^ ILC2s and geometric fluorescence intensity (gMFI) of IL‐5 and IL‐13 (C), and cytokine secretion in the supernatants (D) of ILC2s sorted from Rag2^−/−^ lungs and then cultured in vitro with IL‐33, IL‐2 and IL‐7 for 3 days in the presence or absence of PGE_2_. Data shown as means ± SEMs are from one of two independent experiments. **p < .01 and ***p < .001 by unpaired, 2‐tailed Student's *t*‐test. Veh, vehicle; FSC‐A, forward scatter area. (E) Cell size, ST2 gMFI, and collective percentages of IL‐5^+^IL‐13^−^, IL‐5^+^IL‐13^+^, or IL‐5^−^IL‐13^+^ of ILC2s expanded and cultured with IL‐33, IL‐2 and IL‐7 for 3 days in the presence or absence of indicated reagents. (F) Representative flow cytometric histogram for ST2 expression. *p < .05, **p < .01, ***p < .001, and ****p < .0001 by one‐way ANOVA with post‐hoc Holm–Sidak's multiple comparisons tests. (G and H) Gene enrichment assays (G) and gene expression of pathways of interest (H) in ILC2s cultured with IL‐33 and/or cAMP for 4 h. Raw RNAseq data were retrieved from Gene Expression Omnibus GSE131996, reanalysed and transformed to Z‐scores. NES, normalized enrichment score. (I and J) Expression of c‐Myc and pSTAT5 in ILC2s expanded and cultured with IL‐33, IL‐2 and IL‐7 for 3 days in the presence or absence of indicated reagents. *p* values were calculated by one‐way ANOVA with post‐hoc Holm–Sidak's multiple comparisons tests. (K) Expression of cellular senescence associated genes in ILC2s.

To investigate whether PGE_2_ directly controls ILC2 activation, we sorted ILC2s from lung (Figure 4A–D) and bone marrows using flow cytometry (Figure S8) and cultured them with the cytokine cocktail including IL‐2, IL‐7 and IL‐33 for 3 days. Addition of PGE_2_ significantly decreased ILC2 viability, cellular size [evidenced by reducing forward scatter (FSC)], and IL‐13 production (Figure 4A–E and Figure S8). Activation of the PGE_2_‐EP2/EP4‐cAMP pathway also down‐regulated ST2 expression (Figure 4E,F). In agreement with above results from whole Rag2^−/−^ lung cell culture and a previous report showing that cAMP inhibits ILC2 production of IL‐13 rather than IL‐5,^44^ neither PGE_2_ nor EP agonists inhibited IL‐5 production (Figure 4C–E). These results indicate that PGE_2_ may reduce IL‐13 expression from IL‐5‐ and IL‐13‐double positive cells and turn them into IL‐5‐single positive ILC2s (Figure 4E). In agreement with results from whole Rag2^−/−^ immune cell cultures, EP4 agonist had no effects on IL‐5‐single or IL‐5/IL‐13‐double expressing ILC2s but reduced IL‐13‐single expressing ILC2s (Figure 4E). Other cells such as eosinophils and basophils can also produce IL‐5, which may be the reason for PGE_2_ suppression of IL‐5 production in whole *Rag2* ^*−/−*^ lung cell culture but not in purified ILC2 cultures. ILC2s have been reported produce the regulatory cytokine IL‐10,^45^, ^46^, ^47^ and PGE_2_ promotes IL‐10 production from multiple cell types such as Th2 or macrophages.^10^ We thus examined if PGE_2_ impacted on IL‐10 production from ILC2s. Unlike in other cell types, PGE_2_ still suppressed IL‐10 production from in in vitro cultured ILC2s (Figure S9). Moreover, PGE_2_ inhibition of ILC2 activation and effector function (e.g., ST2 expression and cytokine production) was mimicked by dibutyryl‐cyclic AMP (db‐cAMP, a cell‐permeable cAMP analog) and IBMX (Figure 4E,F). Pre‐treatment of Rp‐8‐CPT‐cAMP, a cAMP antagonist, abrogated PGE_2_ suppression of ST2 expression although Rp‐8‐CPT‐cAMP had no impacts on PGE_2_ inhibition of ILC2 viability and cell size (Figure S10). These results suggest that PGE_2_ directly acts on ILC2s and controls ILC2 survival, growth and function through EP2/EP4 and the downstream cAMP signaling.

### 3.6 cAMP regulates IL‐33‐dependent and ‐independent ILC2 gene expression

To understand the underlying mechanisms for PGE_2_ suppression of ILC2 responses, we examined a published dataset^44^ and analysed the effects of cAMP on ILC2 gene expression. Gene set enrichment analysis showed that, in addition to restricting cell cycle progression as reported previously,^44^ cAMP markedly enhanced apoptosis‐associated pathways (Figure 4G). Expression of pro‐apoptosis associated genes (e.g., *Bad*, *Bak1*, caspases, *Pdcd1*) were upregulated by cAMP independently of IL‐33 (Figure 4H). In contrast, IL‐33‐induced expression of anti‐apoptotic genes (e.g., *Bcl2l1*, *Bcl3*, *Trp53*, *Ptk2*) were down‐regulated by cAMP (Figure 4H). This differential cAMP‐altered expression of pro‐ and anti‐apoptotic genes contributes to overall reduced cell survival. Especially, in consistent to down‐regulation of the hallmark c‐Myc pathway gene expression by cAMP (Figure 4G), our data show that PGE_2_‐cAMP significantly reduced c‐Myc protein expression in ILC2s (Figure 4I).

Moreover, IL‐33 elevated expression of the STAT5 pathway genes including cytokines/receptors (*Il2ra*, *Il9*, *Csf2*, and *Csf2r*), JAK family members (*Jak1* and *Tyk2*) and *Stat5a* and *Stat5b* themselves, which were all down‐regulated by cAMP (Figure 4H). Indeed, PGE_2_ and cAMP restrained STAT5 activation in ILC2s cultured with IL‐2/IL‐7 and IL‐33 (Figure 4J). STAT5 is important for ILC2 homeostasis, accumulation and effector function at lymphoid and nonlymphoid tissues.^48^, ^49^, ^50^ Similar to findings observed in T cells,^51^ cAMP up‐regulated gene expression involved in the IL‐12 pathway (e.g., *Il12rb2*) and the interferon pathway, the latter included cytokine receptors (*Il18r*, *Il18rap*, *Ifng*, *Ifngr1*, *Ifnar1*, and *Ifnar2*) and downstream interferon‐stimulated or gamma‐activated genes (*Ifit1*, *Irf8*, *Isg15*, and *Hif1a*) (Figure 4H). Interferon signaling down‐regulates ILC2 activity and restricts type 2 immune responses.^52^, ^53^ IL‐33 alone had no impacts on IL‐12 and interferon pathway gene expression (Figure 4H). The interferon‐ and IL‐12‐activated STATs (e.g., STAT1/2/4) repress ILC2 activation by antagonizing STAT5.^54^ Similarly, cAMP enhanced STAT3 expression (Figure 4H) which usually competes with STAT5 for binding to gene promoters.^55^ Furthermore, STAT5 is recruited to the c‐Myc enhancer,^56^ which may contribute to cAMP down‐regulation of the MYC pathway (Figure 4G,I). MYC plays an important role in cellular metabolism, and it has been reported to be critical in ILC2‐mediated airway inflammation.^57^ In addition, cAMP upregulated cellular senescence signature genes (*Eomes*, *Foxo1*, *Havcr3*, and *CD244a*) and lymphocyte exhaustion markers (*Tnfrsf18*, *Tigit*, *Ctla4* and *Pdcd1*) but down‐regulated cyclin‐dependent kinase inhibitors (*Cdkn2a* and *Cdkn2b*) (Figure 4K), suggesting that cAMP may foster ILC2 senescence and exhaustion.

### 3.7 PGE_2_ restricts ILC2 cellular metabolism

**FIGURE 5:**
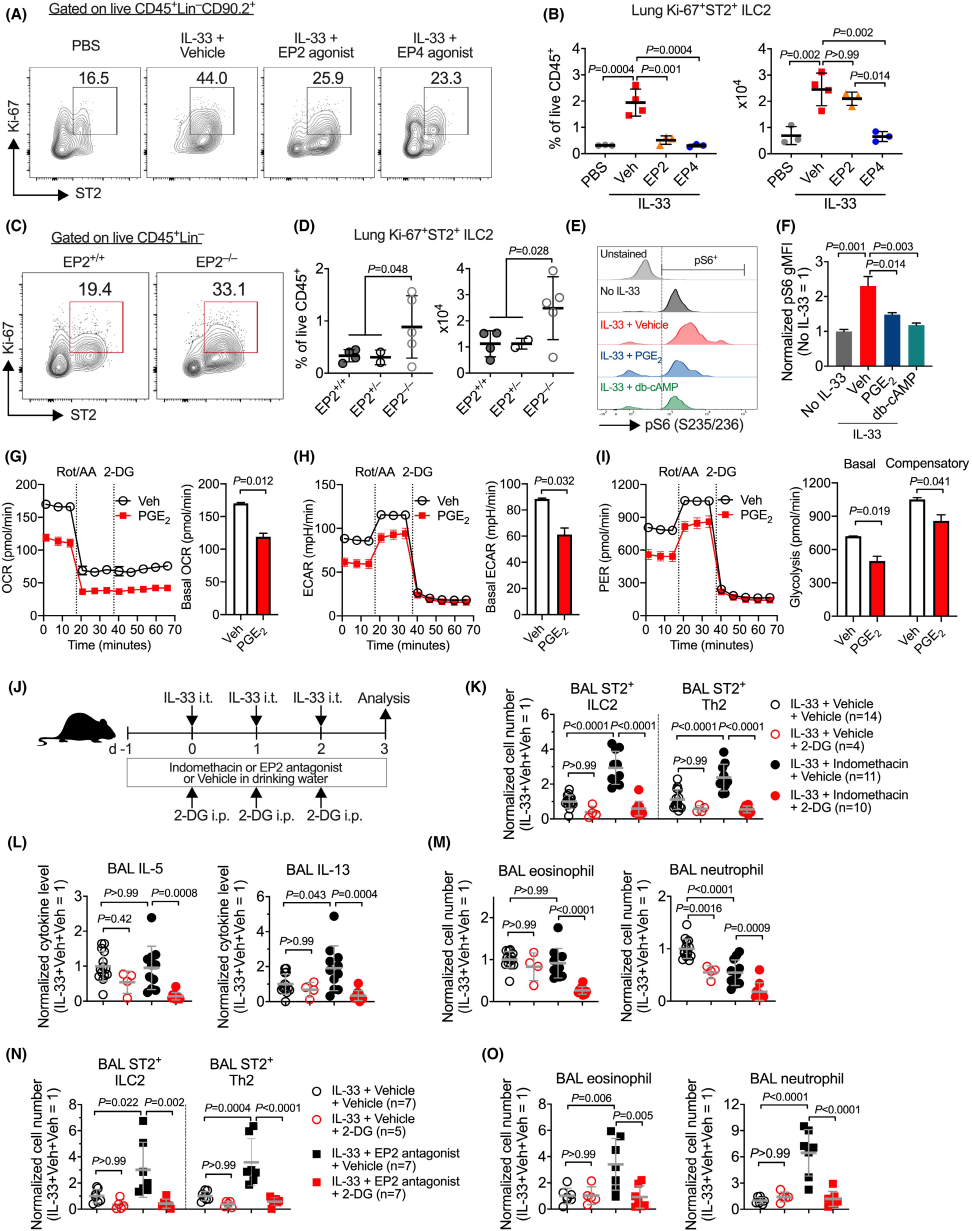
PGE_2_ represses ILC2 cellular metabolism, and blockade of glycolysis attenuates EP2 inhibition‐augmented lung ILC2 responses. (A and B) Representative flow cytometric dot plots (A) and collective percentages and numbers (B) of lung proliferating Ki‐67^+^ ILC2s in C57BL/6 mice administered intratracheally with IL‐33 with PBS (*n* = 3) or IL‐33 with vehicle (*n* = 4), an EP2 agonist (butaprost, *n* = 3), an EP4 agonist (L‐902688, *n* = 3), or both the EP2 and EP4 agonists (*n* = 3) for 3 consecutive days as shown in Figure 3. (C and D) Representative flow cytometric dot plots (C) and collective percentages and numbers (D) of lung proliferating Ki‐67^+^ ILC2s in EP2^+/+^ (*n* = 4), EP2^+/−^ (*n* = 2) or EP2^−/−^ (*n* = 5) mice administered with IL‐33 for 3 consecutive days as shown in Figure 1. (E and F) Representative flow cytometric dot plots (E) and collective expression of pS6 (F) in ILC2s expanded and cultured with IL‐33, IL‐2 and IL‐7 for 3 days in the presence or absence of indicated reagents. (G–I) The oxygen consumption rate (OCR), extracellular acidification rate (ECAR), and proton efflux rate (glycoPER) of ILC2s that were sorted from lungs of Rag2^−/−^ mice and then cultured with IL‐33 with or without PGE_2_ for 3 days. (J) Experimental design. Female C57BL/6 mice were administered intratracheally with IL‐33 with or without 2‐DG for 3 consecutive days and sacrificed 24 h after the last IL‐33 challenge. Mice were also treated with indomethacin, an EP2 antagonist (PF‐04418948) or vehicle control in drinking water from the day before first IL‐33 administration and throughout the experiments. (K–M) Numbers of ST2^+^ ILC2 and ST2^+^ Th2 cells (K), IL‐5 and IL‐13 levels (L), and eosinophil and neutrophil numbers (M) in bronchoalveolar lavages (BAL) from mice treated with IL‐33 with or without indomethacin and 2‐DG. (N and O) Numbers of ST2^+^ ILC2 and ST2^+^ Th2 cells (N) and eosinophils and neutrophils (M) in BAL from mice treated with IL‐33 with or without EP2 antagonist and 2‐DG. Numbers of mice in each group are indicated in the keys. Data shown as means ± SDs (B, D, K–O) or means ± SEMs (F–I) were pooled from one (B, O), two (D) or three (K–M) experiments or representative one of two experiments (E–I). Data in (K–O) were normalized by sexes and presented as fold changes to the IL‐33 + Indomethacin+vehicle group in respective experiments. Each dot in the bar graphs (B, D, K–O) represents one mouse. *p*‐values were calculated by one‐way (B) or two‐way (K–O) ANOVA with post‐hoc Holm–Sidak's multiple comparisons tests or unpaired, 2‐tailed Student's *t*‐tests (D–I).

In this study, we have established that PGE_2_ reduces ILC2 survival, cell size, proliferation, and c‐Myc expression (Figure 4), all are hallmark events of cellular metabolism. Similarly, in vivo activation of EP4 and, possibly EP2, by their respective agonists reduced Ki‐67‐expressing ST2^+^ ILC2s in lungs of mice treated with IL‐33 (Figure 5A,B). In contrast, EP2 deficiency increased Ki‐67^+^ST2^+^ ILC2s in the lung (Figure 5C,D). Furthermore, IL‐33 stimulation induced phosphorylation of the ribosomal protein S6 (pS6), a key signature of cellular metabolic activation. This is again reverted by PGE_2_ and cAMP (Figure 5E,F). These results may imply changes in energy metabolism of ILC2s by PGE_2_‐cAMP signaling. To examine if PGE_2_ directly regulates ILC2 metabolism, we measured metabolic changes in cultured lung ILC2s using the Seahorse assays. Indeed, PGE_2_‐reduced oxygen consumption rate (OCR, an indicator of mitochondrial phosphorylation), extracellular acidification rate (ECAR, an indicator of aerobic glycolysis) and glycolytic proton efflux rate (PER, a real‐time indicator of glycolysis rate) of ILC2s (Figure 5G,H). Furthermore, PGE_2_ suppressed not only basal glycolysis (i.e., physiological mitochondrial respiration) but also compensatory glycolysis when mitochondrial respiration is inhibited (Figure 5I). Thus, our results suggest that PGE_2_ directly inhibits IL‐33‐dependent ILC2 energy metabolism in vitro.

To determine whether PG signaling controls ILC2 responses through inhibiting ILC2 metabolism in vivo, we treated WT C57BL/6 mice with IL‐33 and indomethacin (Figure 5J). Some mice received 2‐deoxy‐D‐glucose (2‐DG), a glucose analogue that inhibits glycolysis through blocking glucose hexokinase. 2‐DG has no impact on IL‐33‐ or helminth infection‐induced ILC2 activation,^58^, ^59^ but it reduces ILC2 responses when ILC2s are over‐activated such as in PD‐1‐deficient mice.^59^ In agreement with these previous reports,^58^, ^59^ 2‐DG affected neither IL‐33‐induced ILC2 accumulation and type 2 cytokine production nor eosinophil recruitment in mice with intact PG signaling (i.e., without indomethacin‐treatment) (Figure 5K–M). However, in indomethacin‐treated mice where biosynthesis of endogenous PGs including PGE_2_ was blocked, 2‐DG strikingly reduced ILC2 and Th2 numbers and type 2 cytokine production in the airway (Figure 5K,L). Accordingly, overall lung inflammation was also decreased by 2‐DG in indomethacin‐treated mice as evidenced by reduction of both eosinophils and neutrophils in the BAL fluid (Figure 5M). 2‐DG did not affect lung ILC2 numbers nor cytokine production in both indomethacin‐ or vehicle‐treated mice except the reduction of lung eosinophils in mice treated with indomethacin but not vehicle control (Figure S11). As indomethacin inhibits production of all PGs, we wondered if PGE_2_, especially the EP2 receptor, inhibits lung ILC2 responses through inhibiting ILC2 metabolism. To address this, we treated mice with PF‐04418948, a highly selective EP2 antagonist,^60^ with or without 2‐DG (Figure 5J). In agreement with our observations in EP2KO mice, the EP2 antagonist also significantly increased numbers of ILC2s, Th2 cells, eosinophils and neutrophils in the BAL fluid (Figure 5N,O). Similar to findings in mice treated with indomethacin, augmented ILC2 responses in EP2 antagonist‐treated mice were also markedly reduced by 2‐DG although it remained no effects on ILC2 responses in mice treated with vehicle control (Figure 5N,O). Taken together, these results suggest that PGE_2_ impedes ILC2 energy metabolism and constrains IL‐33‐induced lung type 2 immune responses.

## 4 DISCUSSION

Here, we report that PGE_2_‐EP2/EP4 signaling limits type 2 lung inflammation through negative regulation of ILC2 responses. We have shown that deficiency of EP2, but not EP4, enhanced lung ILC2 responses, suggesting that endogenous ligands (such as PGE_2_ and others such as PGE_1_) more preferentially bind to EP2 than EP4. Although both EP2 and EP4 activate the same downstream cAMP‐PKA signaling pathway, they can have redundant or additive functions in vivo. For example, EP2 and EP4 collaboratively promotes T cell‐mediated psoriasis.^61^ Under certain circumstances, the in vivo actions of PGE_2_ may be dominantly mediated by one receptor, leaving the other dispensable. For example, the effects of PGE_2_ on intestinal homeostasis are largely mediated by EP4, and EP2 exerts few effects.^62^, ^63^

However, exogenous administration of both EP2 and EP4 agonists effectively inhibited lung ILC2 responses, suggesting that activation of both receptors have similar biological effects in terms of ILC2 suppression. The distinct effects of EP2 and EP4 may be due to different receptor binding affinity by natural and synthetic ligands. Indeed, endogenous PGE_2_ or PGE_1_ have a Ki value of ~10 nM for EP2 binding, while the commonly‐used EP2 selective agonist, butaprost, has a Ki value of ~100 nM to bind EP2,^64^, ^65^ thus 10‐fold lower affinity than PGE_2_. Consequently, administration of butaprost did not inhibit lung ILC2 responses (data not shown). Another selective EP2 agonist, butaprost (free acid) that were used in this study, binds to EP2 with 10–100 times higher the affinity than butaprost.^66^ On the other side, the selective EP4 agonist L‐902688 (Ki value of ~0.4 nM) has about 5‐fold higher affinity to bind EP4 than PGE_2_ (Ki value of ~2 nM).^65^, ^67^ Therefore, although endogenous PGE_2_ and/or PGE_1_ preferentially stimulate EP2, exogenous ligands take advantages of their higher selectivity to activate respective cognate receptors.

Our results may also indicate that in the lung, EP2 may have already been endogenously activated at the optimal level for repressing ILC2 responses. Thus, further activation of EP2 requires stronger stimuli, for example using an agonist with higher EP2 affinity like butaprost (free acid) at relatively higher dosages to achieve satisfactory efficiency in vivo. On the other side, EP4 signaling does not sound to be properly activated by endogenous ligands, thus activation of this receptor by an exogenous agonist results in considerable effects. In addition, the discrepancy in effects between EP4 agonism and EP4 deficiency on regulation of lung ILC2 responses may also indicate that EP4 expression on different lung cell types has distinct effects during type 2 inflammation. For example, PGE_2_ may affect T cell functions that counteract PGE_2_ inhibition of ILC2s. Moreover, the strength (e.g., the amounts of local lung resident PGE_2_) and the timing of endogenous versus exogenous EP4 activation in the course of lung allergic inflammation may also influence the overall effects of EP4 on ILC2 responses. Further studies are needed to finely examine these possibilities.

PGE_2_ has been indicated to be associated with various lung conditions including neutrophilic inflammation,^22^ infections (e.g., mycobacterium tuberculosis^20^) and COPD.^24^ Blocking synthesis of PGE_2_ and other PGs by NSAIDs like indomethacin may be beneficial for treating these conditions. It is worth to note that indomethacin was reported to bind to mouse CRTH2,^68^ a receptor for prostaglandin D_2_ that mediates ILC2 migration.^15^, ^69^ It was suggested that CRTH2 mediates ILC2 recruitment to the lung when IL‐33 is administered systemically (e.g., via intraperitoneal injection), but it has few effects on ILC2 migration to the lung if IL‐33 is administered directly to the lung.^16^ In our studies, we intratracheally administered IL‐33 into the lung, thus the enhancement of lung ILC2 responses by indomethacin is most likely through inhibition of endogenous PGs. Furthermore, our findings that EP2 rather than EP4 is the dominant mediator of endogenous PGE_2_ effects on the regulation of lung ILC2 responses specifically reflect the findings from clinical studies. Indeed, *PTGER2* gene polymorphisms are associated with asthma especially NERD and patients with NERD have decreased EP2 expression.^14^, ^19^, ^20^ Our results suggest that the down‐regulation or alteration of EP2 signaling in NERD patients may contribute to augmented ILC2 responses and inflammation. Thus, pharmacological activation of EP4 may be a promising therapeutic approach to limit augmented ILC2 responses in asthma patients with low EP2 expression or function.

We have also reported that PGE_2_ through activation of cAMP suppresses ILC2 energy metabolism, which controls cell survival, proliferation, activation and effector function. Besides PGE_2_, many molecules that can activate the cAMP pathway such as PGI_2,_ ^17^ β2‐adrenergic receptor agonist (e.g., norepinephrine),^70^ and calca‐encoding calcitonin gene‐related peptide^44^, ^71^, ^72^ have all been found to suppress ILC2 responses and allergic lung inflammation. Our findings of regulation of ILC2 gene expression by the PGE_2_‐cAMP pathway via IL‐33‐dependent and ‐independent mechanisms suggest that PGE_2_ may have broad impacts on ILC2‐mediated type 2 immune responses. It is worth noting that although cAMP represses ILC2 activation and IL‐13 production, it does not inhibit IL‐5 expression, which was in line with the findings of other studies.^44^ Indeed, cAMP was reported to mediate elevation of IL‐5 production from homeostatic ILC2s.^73^ Actually, PGE_2_‐cAMP signaling suppresses IL‐5 production from IL‐5/IL‐13‐co‐expressed ILC2s. Thus, it may be worth investigating for the future if PGE_2_‐cAMP activates distinct downstream pathways in different ILC2 subsets, for example taking advantage of single cell RNA‐sequencing.

Activation of the energy metabolic programs is essential for ILC2 survival, proliferation and effector function as blockade of mTORC1 signaling by rapamycin inhibits IL‐33‐induced ILC2 responses and airway inflammation.^74^ Our observation that blocking glycolysis by 2‐DG has no effect on IL‐33‐ or parasite‐mediated ILC2 activation at the ‘normal’ level has also been reported by other groups,^58^, ^59^ but it impairs excessive ILC2 responses when ILC2s are ‘overactivated’, for example, by removing ‘endogenous inhibitors’ such as PD‐1,^59^ PGE_2_ or EP2. This is probably because normally activated ILC2 had low‐gene expression of glycolysis‐related enzymes,^75^ but overactivated ILC2s have enhanced expression of those enzymes to meet the increased requirement of energy consumption. Thus, blockade of glycolysis can repress excessive ILC2 responses, for example, in asthma and NERD.

This current research has several limitations. First, our results clearly indicated that PGE2‐EP2 signaling directly suppresses lung ILC2 responses in viitro and in vivo using global EP2‐deficient mice. However, due to the lack of ILC2‐specific EP2‐deficient mouse lines, we could not address whether ILC2‐specific EP2 signalling inhibits lung ILC2 responses and, if yes, whether this is dependent of its inhibition of ILC2 cellular metabolism in vivo. Second, as limited access to key resources (especially for an appropriate seahorse machine), we could not directly check if PGE_2_ inhibits ILC2 oxygen consumption and glycolysis through EP2 and EP4. Third, we did not examine if PGE_2_ suppression of lung ILC2 responses contributes to human asthma and NERD, which is desired to investigate following this current research.

Our current work has significantly improved our understanding of control of lung allergic responses. First, we have demonstrated that blockade or deficiency of endogenous EP2 signaling augments lung ILC2 responses and eosinophilic inflammation. This is in agreement with published findings from humans that down‐regulation of the PGE_2_‐EP2 pathway is a feature of NRED.^14^, ^19^, ^20^ Second, our results using global and ILC2/Th2‐conditional knockout mouse lines clearly showed that while endogenous EP4 signaling is dispensable for control of lung ILC2 responses, EP4 agonism markedly reduced IL‐33‐dependent lung ILC2 responses. Thus, exogenous activation of EP4 could therapeutically attenuate ILC2‐dependent lung allergic reactions, especially in asthma patients where PGE_2_‐EP2 signaling is down‐regulated. Third, our work suggests that PGE_2_ curtails ILC2 cellular metabolism through activating the EP2/EP4/cAMP signalling. More importantly, our findings that in vivo inhibition of glycolysis successfully reduces lung ILC2 responses augmented by diminishing PGE_2_‐EP2 signaling suggest a potential strategy for treatment of NERD that has down‐regulated EP2 signaling and enhanced ILC2 responses.

Current treatments do not provide a cure for asthma and in the case of NERD patients, such treatments may not be effective. Our work highlights the EP4‐cAMP pathway as a potential therapeutic target for asthma and in particular for NERD, where small molecules that harness activation of EP4 and/or elevation of intracellular cAMP levels could be of clinical benefit. Our results show that use of phosphodiesterase inhibitor effectively inhibits ILC2 responses in vitro and in vivo, supporting the development of phosphodiesterase inhibitors as considered for treatment of asthma and other lung diseases.^76^, ^77^ Furthermore, we propose that targeting ILC2 energy metabolic pathways (e.g., glycolysis) may be beneficial in the control of NSAID‐dependent augmentation of type 2 lung inflammation in patients with NERD.

## AUTHOR CONTRIBUTIONS

CTR and CY conceived of all the experiments. CTR, JMF, MG, and PJ performed the experimental work with help from RD, SM, and CY. YZ and BZ analysed RNAseq data. DJS, RMB, SN, HJM, RMM, JKJS, and AGR provided essential animal lines, key reagents and critical inputs. The manuscript was written by CTR and CY, with critical input from YZ, RMB, SN, HJM, RMM, JKJS, and AGR. This project was managed and supervised by CY.

## FUNDING INFORMATION

This work is supported in part by UKRI Medical Research Council (MR/R008167/1 to C.Y.), Cancer Research UK (C63480/A25246 to C.Y.). M.G. received a CMVM Research Adaptation Funding support.

## CONFLICT OF INTEREST

The authors have no conflict of interest.

## Supporting information


Figs S1–S11.
