# Supplementary material for: Metabolic regulation by prostaglandin E2 impairs lung group 2 innate lymphoid cell responses
Source: Allergy. 2022 Oct 14;78(3):714–30. doi: 10.1111/all.15541 (PMC10952163; doi:10.1111/all.15541)
Supplement: Supplementary file 1 — Figs S1–S11. [file ALL-78-714-s001.docx]

Supplementary Materials

**Metabolic regulation by prostaglandin E_2_ impairs lung group 2 innate lymphoid cell responses**

Calum T. Robb,^1^ You Zhou,^2^ Jennifer M. Felton,^1^ Birong Zhang,^2^ Marie Goepp,^1^ Privjyot Jheeta,^1^ Danielle J. Smyth,^3^ Rodger Duffin,^1^ Sonja Vermeren,^1^ Richard M. Breyer,^4^ Shuh Narumiya,^5^ Henry J. McSorley,^3^ Rick M. Maizels,^6^ Jürgen K.J. Schwarze,^1^ Adriano G. Rossi,^1^ Chengcan Yao,^1^


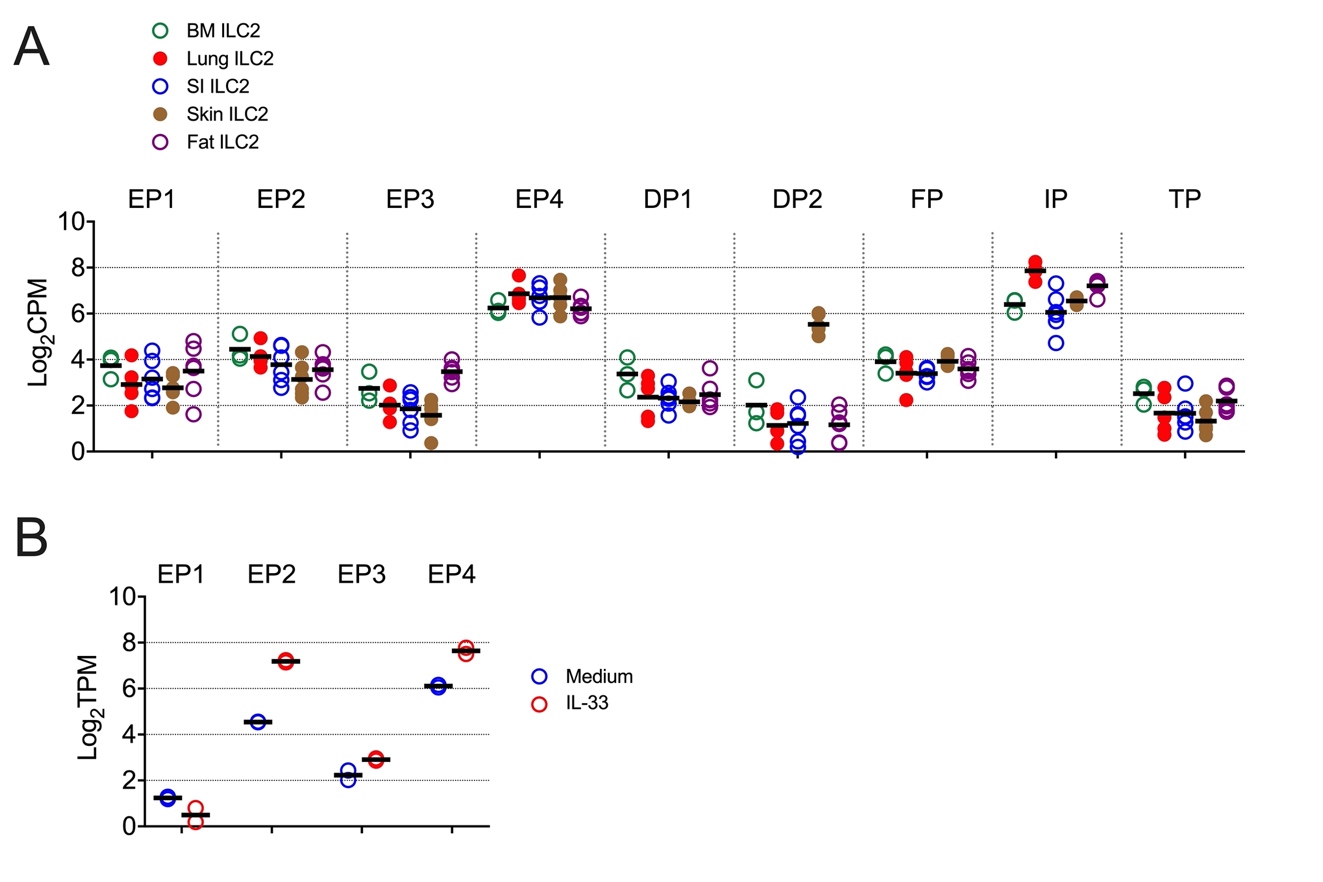


**Supplementary Figure 1. (A)** Gene expression of prostaglandin receptors on naïve ILC2s isolated from various tissues. Raw bulk RNAseq data was retrieved from Gene Expression Omnibus GSE117470. (B) Gene expression of PGE_2_ receptors on lung ILC2s cultured with or without IL-33 for 24 h. Raw bulk RNAseq data was retrieved from Gene Expression Omnibus GSE131996.


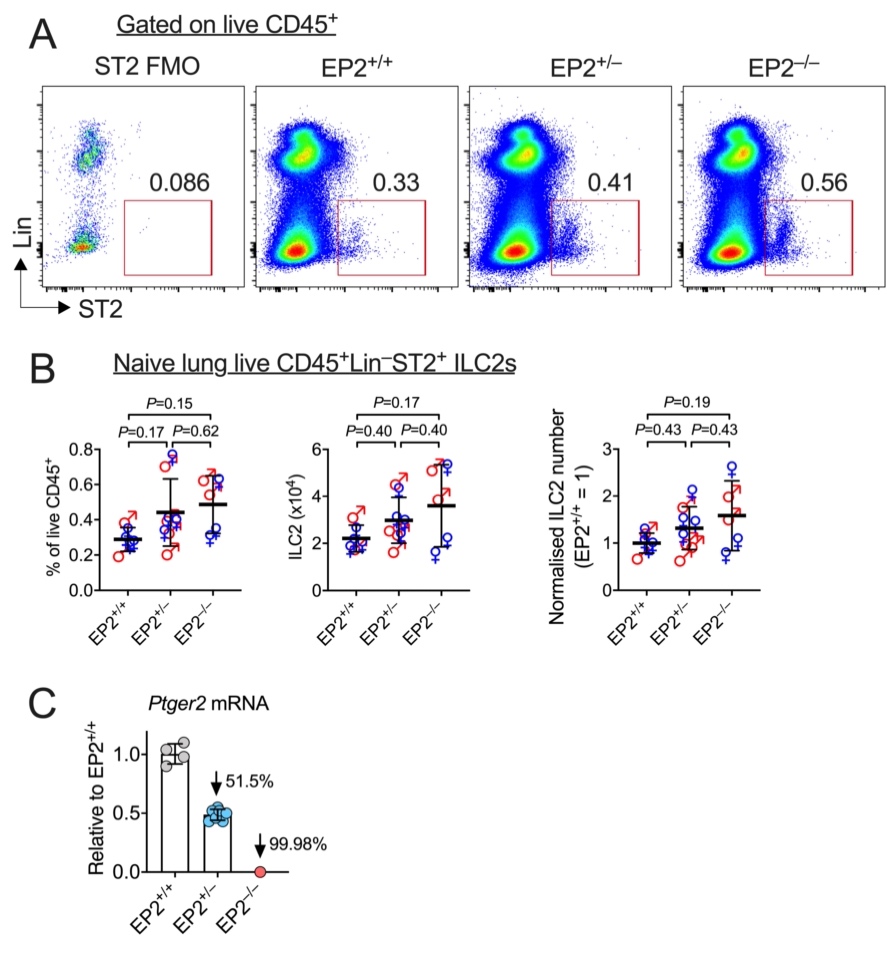


**Supplementary Figure 2. EP2 deficiency does not affect lung ILC2s. (A and B)** Representative flow cytometric dot plots **(A)** and collective percentages **(B)** of lung ILC2s from naïve EP2^+/+^, EP2^+/–^ and EP2^–/–^ mice. Cells in **(A)** were pre-gated on live CD45^+^ immune cells. Each dot in the bar graphs represents one mouse. *P* values were calculated by one-way ANOVA with post-hoc Holm-Sidak's multiple comparisons tests. **(C)** *Ptger2* (EP2) gene expression on spleen immune cells from EP2 wildtype, heterozygous or homozygous mice. *Ptger2* gene expression detected by real-time RT-PCR and normalised to EP2^+/+^ cells. *Ptger2* gene is not expressed in EP2^–/–^ cells and reduced by half in EP2^+/–^ cells compared to EP2^+/+^ cells.


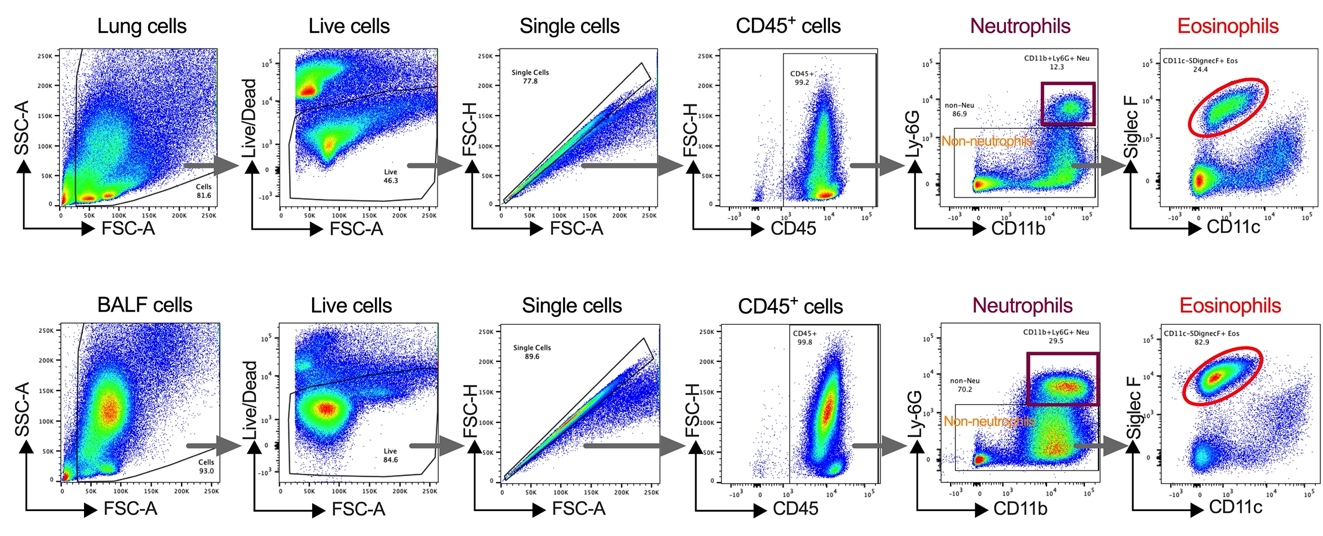


**Supplementary Figure 3. Gating strategies for lung and BAL neutrophils and eosinophils.**


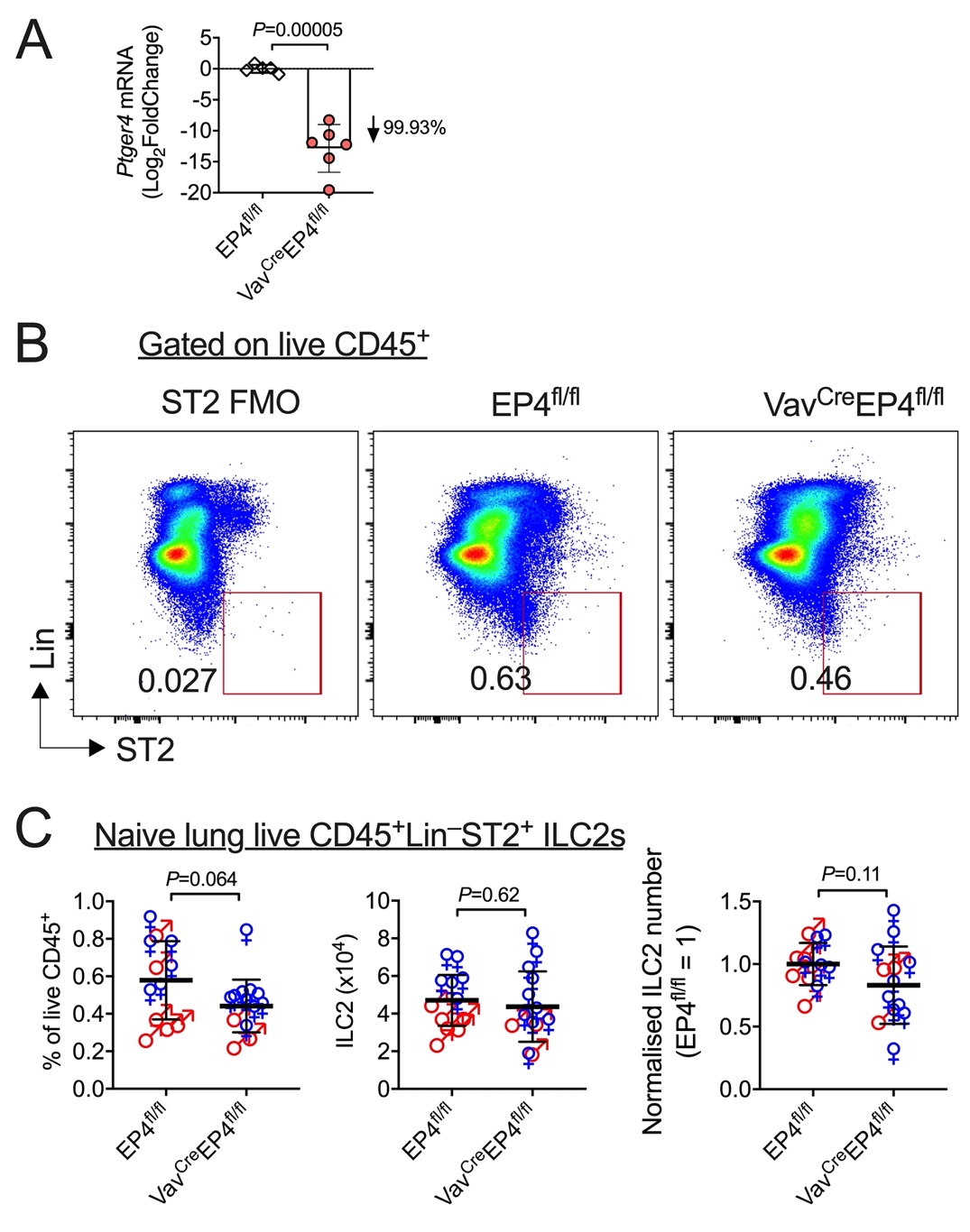


**Supplementary Figure 4. EP4 deficiency does not affect lung ILC2s. (A)** *Ptger4* (EP4) gene expression on bone marrow CD90.2^+^ immune cells (purified by mouse CD90.2 positive selection kit, STEMCELL Technologies) from EP4^fl/fl^ or Vav^Cre^EP4^fl/fl^ mice. Ptger4 gene expression was detected by real-time RT-PCR and presented as log2FoldChange compared to EP4^fl/fl^ cells. **(B and C)** Representative flow cytometric dot plots **(B)** and collective percentages **(C)** of lung ILC2s from naïve EP4^fl/fl^ and Vav^Cre^EP4^fl/fl^ mice. Cells in **(B)** were pre-gated on live CD45^+^ immune cells. *P* values were calculated by unpaired two-tailed *t*-test.


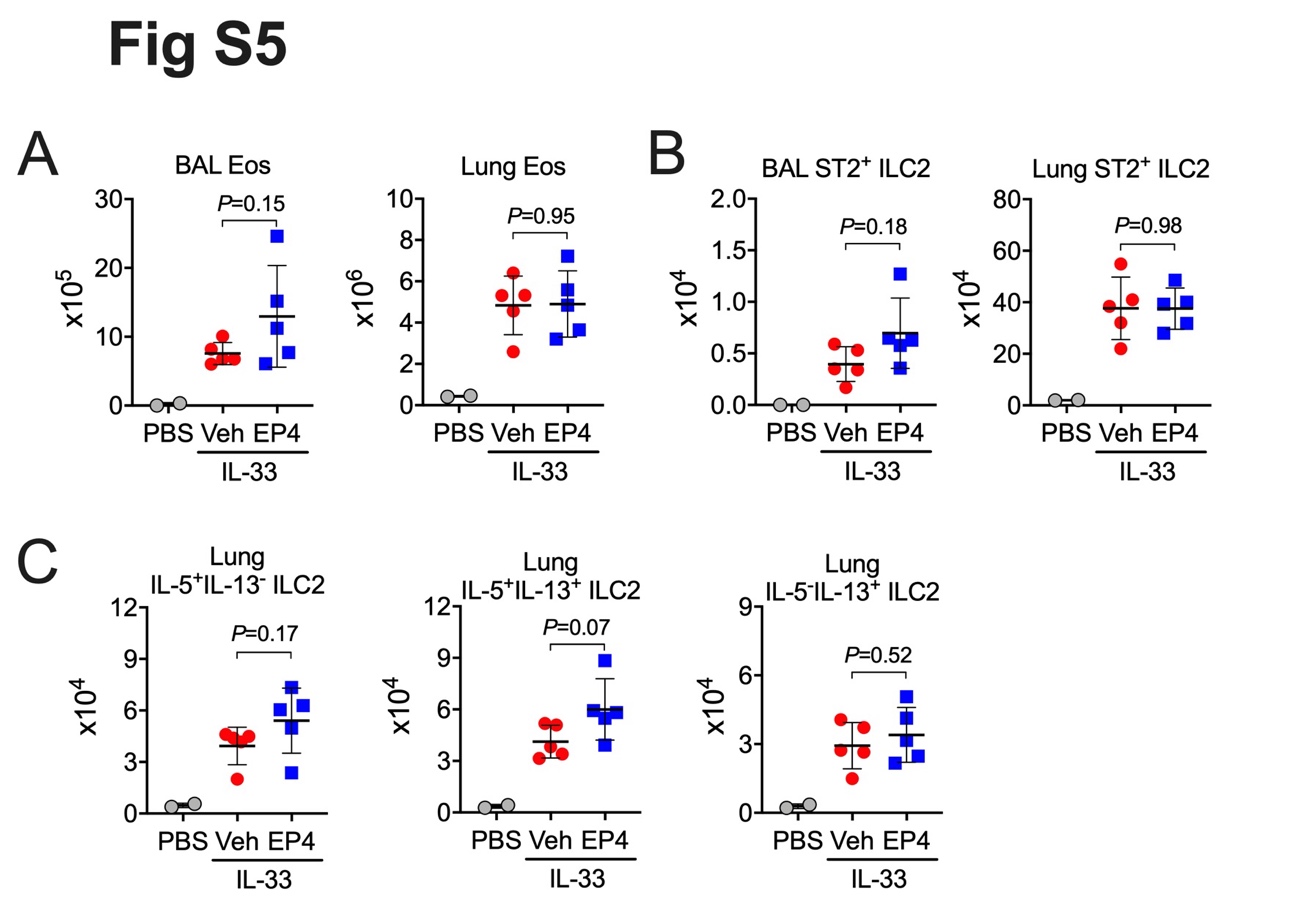


**Supplementary Figure 5. L-902,688 does not affect EP4KO lung ILC2 responses.** EP4KO mice were administered intratracheally with PBS (n=2) or IL-33 with vehicle (n=5) or EP4 agonist (L-902,688, n=5) 3 consecutive days, and sacrificed 24 h after the last IL-33 challenge. **(A)** Eosinophils in the lungs and the bronchoalveolar lavages (BAL) fluids. **(B)** ST2^+^ total ILC2 numbers in the BAL fluids and lungs. **(C)** Numbers of IL-5- or IL-13-expressing ILC2s in the lungs. Data were normalised to the IL-33 plus Vehicle groups in respective experiments and sexes and shown as means ± SDs. Each dot in the bar graphs represents one mouse. *P* values were calculated by unpaired, 2-tailed *t*-tests.


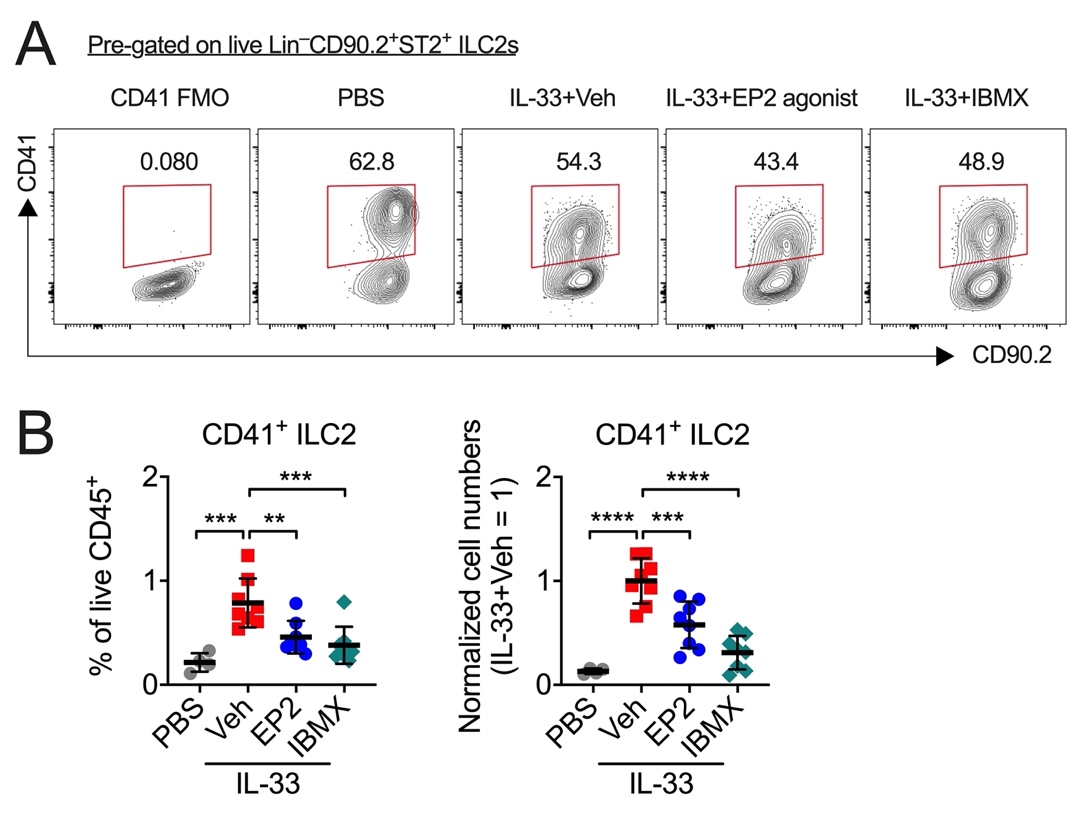


**Supplementary Figure 6. Effects of EP2 agonism and PDE inhibition on lung platelet-adherent ILC2s.** Female C57BL/6 mice were administered intratracheally with PBS or IL-33 with vehicle, EP2 agonist [butaprost (free acid)], EP4 agonist (L-902,688), or IBMX for 3 consecutive days, and sacrificed 24 h after the last IL-33 challenge. Representative flow cytometric dot plots **(A)** and collective numbers **(B)** of lineage(CD3/CD19/CD11c/CD11b/NK1.1/CD200R3)^–^CD90.2^+^ST2^+^CD41^+^ platelet-adherent ILC2s in the lung. Cells in **(A)** were pre-gated on the live Lin–CD45^+^CD90.2^+^ST2^+^ ILC2s. Data shown as means ± SDs were pooled from two independent experiments. Each dot in the bar graphs represents one mouse. ***P*<0.01, ****P*<0.001, *****P*<0.0001 by one-way ANOVA with post-hoc Holm-Sidak's multiple comparisons tests.


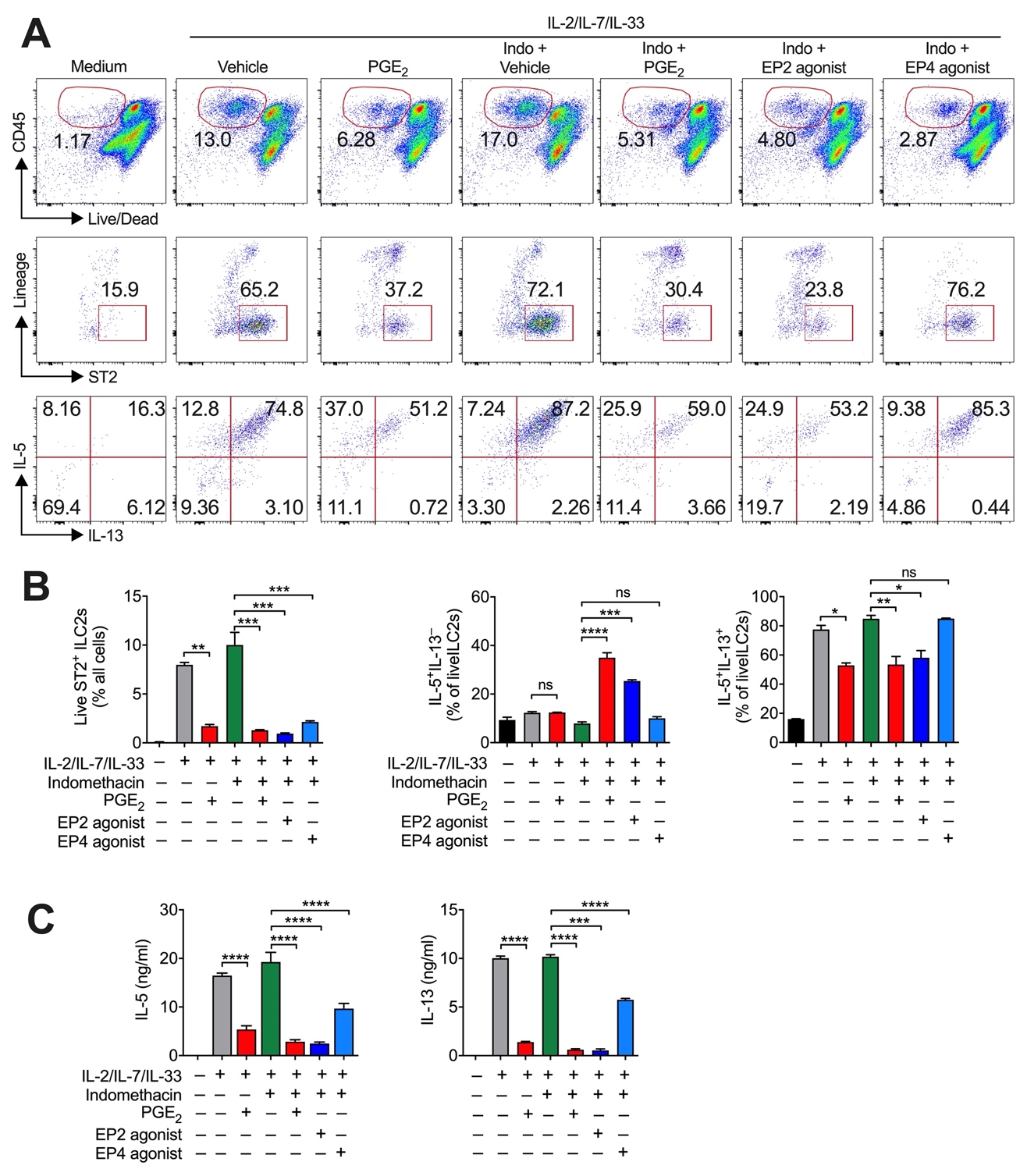


**Supplementary Figure 7. Effects of PGE_2_ on lung ILC2 responses *in vitro*.** Whole populations of single cells isolated from Rag2^-/-^ lungs were cultured with IL-33, IL-2 and IL-7 in the presence or absence of indomethacin (Indo), PGE_2_, EP2 agonist or EP4 agonist for 3 days. **(A)** Representative flow cytometric dot plots. **(B)** Collective percentages of live CD45^+^Lin^–^ST2^+^ total ILC2s or IL-5/IL-13-expressing ILC2s. **(C)** IL-5 and IL-13 levels in the supernatants after 3 days of ILC2 culture. Data shown as means ± SEMs are one from 2 experiments. **P*<0.05, ***P*<0.01, ****P*<0.001, *****P*<0.0001 by one-way ANOVA with post-hoc Holm-Sidak's multiple comparisons tests.


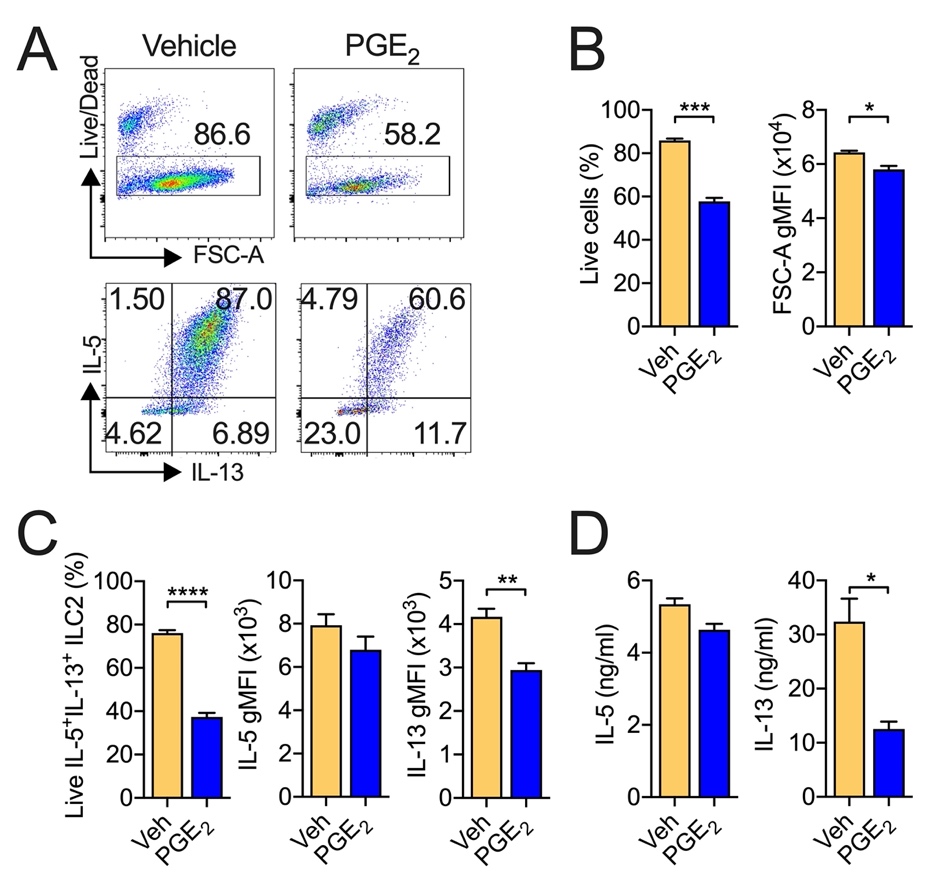


**Supplementary Figure 8. Effects of PGE_2_ on bone marrow ILC2 responses *in vitro*.** ILC2s were sorted from Rag2^-/-^ bone marrow cells and cultured with IL-33, IL-2 and IL-7 in the presence or absence of PGE_2_ for 3 days. **(A)** Representative flow cytometric dot plots. **(B)** Viability and cell size (indicated by FSC-A gMFI). **(C)** Percentages of live IL-5^+^IL-13^+^ ILC2s and gMFI for IL-5 and IL-13. **(D)** Cytokine secretion in the cell culture supernatants. Data shown as means ± SEMs are from one of two independent experiments. **P*<0.05, ***P*<0.01, ****P*<0.001, *****P*<0.0001 by unpaired, 2-tailed Student *t*-tests. Veh, vehicle.


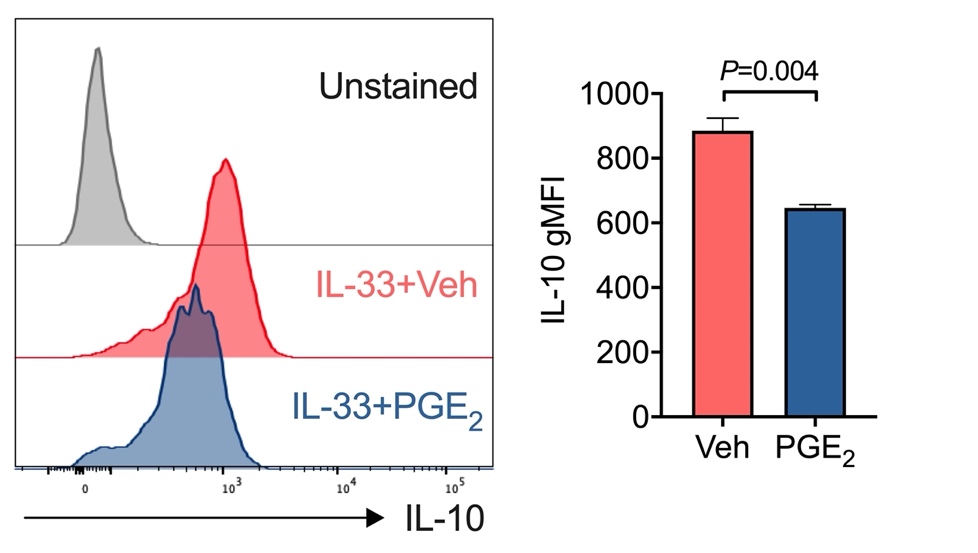


**Supplementary Figure 9. Effects of PGE_2_ on IL-10 expression in ILC2s.** ILC2s were sorted from Rag2^-/-^ mice and cultured with IL-33, IL-2 and IL-7 for 3 days in the presence or absence of PGE_2_. IL-10 was detected by flow cytometry. *P* value was calculated by unpaired, 2-tailed Student *t*-tests. Veh, vehicle.


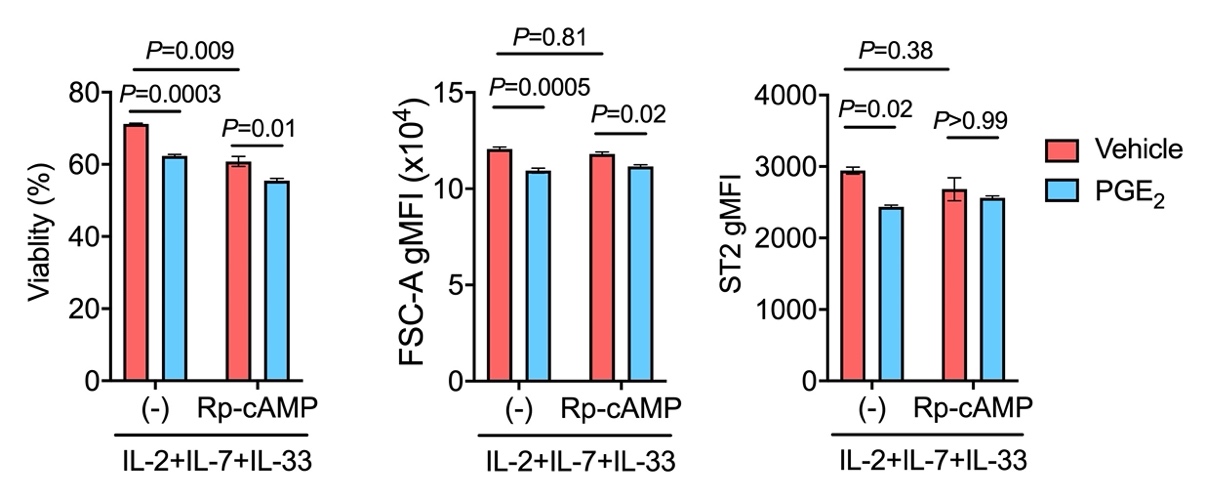


**Supplementary Figure 10. Effects of Rp-8-CPT-cAMP on ILC2 activation *in vitro*.** ILC2s were sorted from Rag2^-/-^ mice and cultured with IL-33, IL-2 and IL-7 for 3 days in the presence or absence of PGE_2_ and/or Rp-8-CPT-cAMP (Rp-cAMP). Cell viability, FSC and ST2 expression were detected by flow cytometry. Data shown as means ± SEMs are from one of two independent experiments. P values were calculated by two-way ANOVA with post-hoc Holm-Sidak’s multiple comparisons tests.


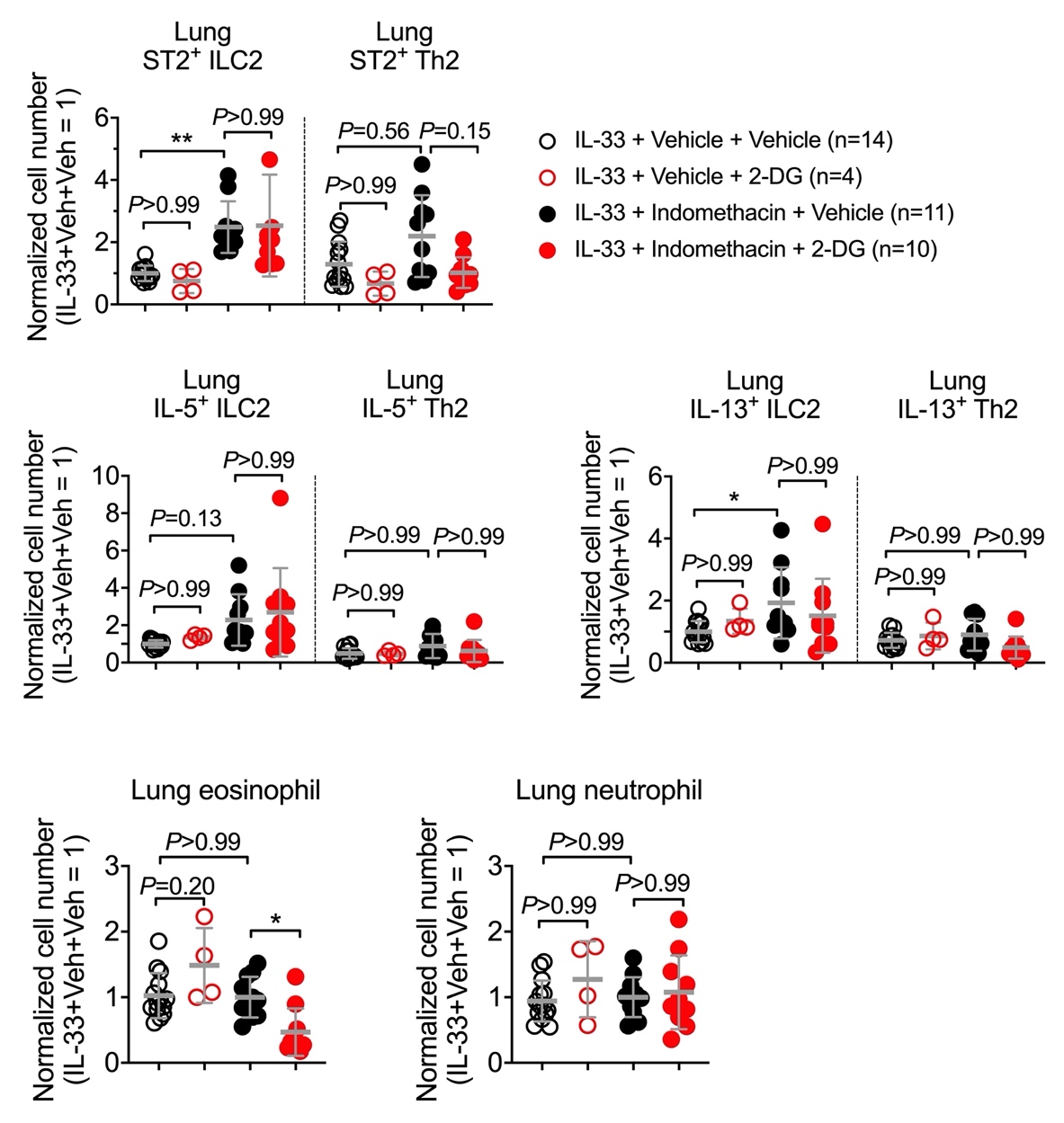


**Supplementary Figure 11. Effects of 2-DG on lung ILC2s.** Female C57BL/6 mice were administered intratracheally with IL-33 with or without 2-DG for 3 consecutive days and sacrificed 24 h after the last IL-33 challenge as in Fig 5J. Mice were also treated with indomethacin or control in drinking water from the day before first IL-33 administration and throughout the experiments. Numbers of lung ST2^+^, IL-5^+^ or IL-13^+^ ILC2s and Th2 cells as well as eosinophil and neutrophil numbers. Data shown as means ± SDs were pooled three experiments. Data were normalized as fold changes to the IL-33+Indomethacin+vehicle group in respective experiments. Each dot in the bar graphs represents one mouse. **P*<0.05, ***P*<0.01 by two-way ANOVA with post-hoc Holm-Sidak's multiple comparisons tests.
